# DNA/RNA Hybrid Hairpin Gold Nanobeacons Targeting miR-31 Reprogram Invasion in Lung Cancer and Remodel Tumor Histo-Architecture *In Vivo*

**DOI:** 10.1021/acsmaterialsau.5c00231

**Published:** 2026-04-07

**Authors:** Diana P. Sousa, Jhenifer Oliveira, Catarina F. Martins, João Conde

**Affiliations:** † NOVA Medical School|Faculdade de Ciências Médicas, NMS|FCM, Universidade NOVA de Lisboa, 1169-056 Lisbon, Portugal; ‡ Comprehensive Health Research Centre (CHRC), NOVA Medical School|Faculdade de Ciências Médicas, NMS|FCM, Universidade NOVA de Lisboa, 1169-056 Lisbon, Portugal

**Keywords:** gold nanoparticle, molecular beacon, microRNA, lung cancer, invasion, endosomal escape

## Abstract

Readout-enabled delivery of anti-microRNA (miRNA) therapeutics remains a bottleneck for translating RNA nanomedicine against invasion and metastasis. We report ∼60 nm PEG-gold nanobeacons bearing a Cy3-labeled anti-miR-31-5p hairpin and establish a chemistry-to-phenotype pipeline spanning physicochemical control, cellular trafficking, functional phenotyping, miR-31 kinetics, transcriptomics, and histology-only *in vivo* analysis. The formulation exhibits invariant plasmonics, stepwise growth in hydrodynamic diameter with low dispersity, and moderated ζ-potential, confirming a stable, readout-enabled architecture. In lung cancer cells with high endogenous miR-31, nanobeacons are efficiently internalized, traffic predominantly to lysosomes, and selectively suppress 3D invasion at 10–20 nM while leaving 2D migration and viability unchanged. A low-miR-31 cell line shows minimal response, supporting target dependence. Time-course qRT-PCR reveals a delayed decrease in mature miR-31 (120–144 h) consistent with a two-phase mechanism: early functional sequestration by a minority cytosolic fraction followed by transcriptional down-tuning. Bulk RNA-seq at 72 h captures early remodeling of adhesion/ECM, integrin/TGF-β/WNT, and cytokine programs with directionality consistent with derepression of the miR-31 regulon. In xenografts locally exposed to nanobeacons, histology shows intratumoral Cy3-positive clusters, innate-immune-like infiltrates, focal reductions in *K*_i_-67, fibrotic stroma, increased necrosis and inflammation, spatial evidence of local microenvironment remodeling without invoking tumor-size end points. Together, these results link precise materials design to a target-engaged, noncytotoxic anti-invasive outcome and define actionable levers, especially enhanced endosomal escape and pathway-level validation, to advance readout-enabled miRNA nanotherapeutics toward translation.

## Graphical abstract

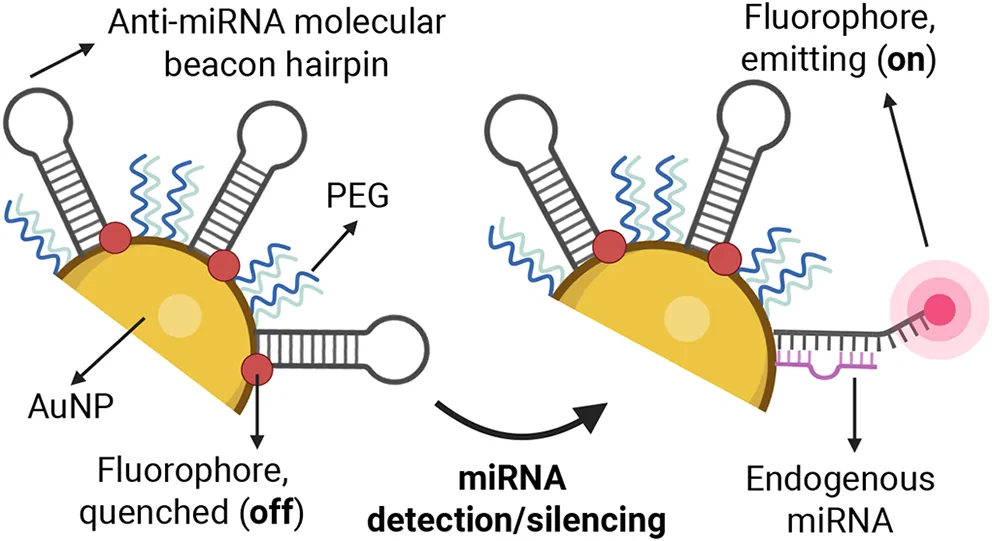

## Introduction

Cancer invasion and metastasis remain the principal causes of cancer mortality and are driven by coordinated rewiring of cytoskeletal dynamics, cell–cell and cell-matrix adhesions, extracellular matrix (ECM) remodeling, and stromal crosstalk.^1,2^ MicroRNAs (miRNAs) occupy a privileged layer in this hierarchy by tuning entire gene networks with modest yet combinatorial effects on translation and mRNA stability^3,4^ (Scheme 1A). Among these, miR-31 has emerged as a context-dependent regulator of epithelial plasticity whose activity interfaces with focal adhesion turnover, Rho GTPase signaling, integrin trafficking, cadherin switching, and matrix metalloproteinase (MMP) activity, pathways that converge on three-dimensional (3D) invasion programs.^5–8^

**Scheme 1:**
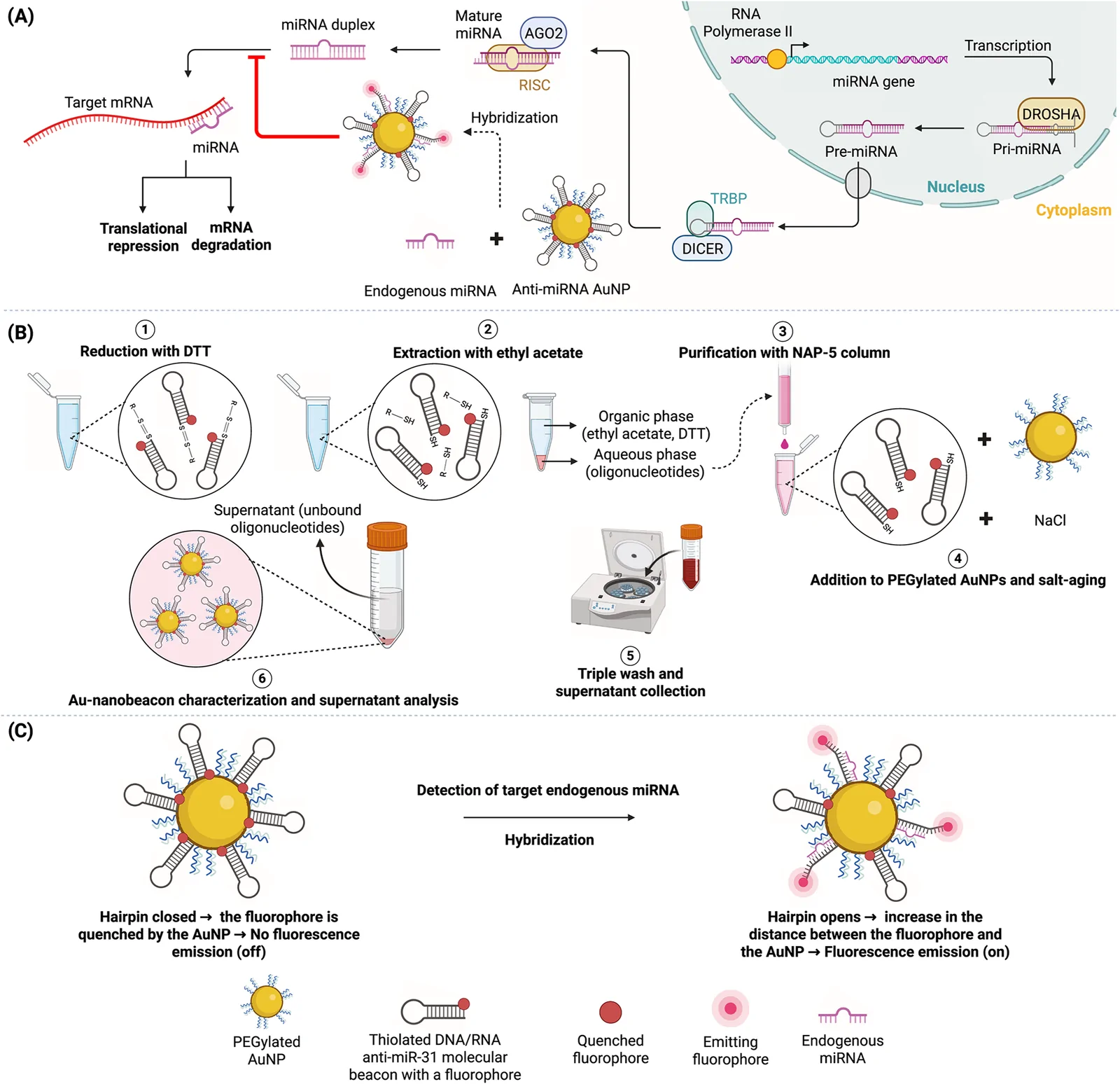
a (A) miRNA biosynthesis and inhibition of miRNA actions by addition of anti-miRNA nanobeacons. (B) Preparation of gold nanobeacons: oligonucleotides react with DTT to reduce disulfide bridges (1) and are then purified via organic extraction (2) and NAP-5 columns (3). Purified oligonucleotides are added to PEGylated AuNPs and subjected to salt-aging (4), being then washed to remove unbound oligonucleotides (5). Finally, the obtained gold nanobeacons are characterized (6). (C) Anti-miRNA gold nanobeacon system, and how it works: a PEGylated AuNP with thiolated DNA/RNA anti-miR-31 molecular beacons carrying a fluorophore that is quenched by the AuNP (hairpin closed, “off”; left). Upon detection of the endogenous target miRNA (miR-31), the hairpin opens and the fluorophore emits, due to the increase in the distance from the AuNP (“on”, right). Created in BioRender. BioRender User (2026) https://eur01.safelinks.protection.outlook.com/?url=https%3A%2F%2Fbiorender.com%2Fe54029k&data=05%7C02%7Cjoao.conde%40nms.unl.pt%7Cf575755f3f08453ddaf108de6365ffa4%7C122d98e12cef4577916ba43bb901dd51%7C0%7C0%7C639057486912089718%7CUnknown%7CTWFpbGZsb3d8eyJFbXB0eU1hcGkiOnRydWUsIlYiOiIwLjAuMDAwMCIsIlAiOiJXaW4zMiIsIkFOIjoiTWFpbCIsIldUIjoyfQ%3D%3D%7C0%7C%7C%7C&sdata=4yo90tdIHLJVq9nSMg0doMe7FdY%2FRfLKDF4iMu03070%3D&reserved=0

Across lung cancer models, miR-31 expression is frequently elevated in aggressive phenotypes and correlates with invasive behavior.^9–11^ Mechanistically, miR-31 targets a distributed set of regulators including (but not limited to) RASA1 and other Ras/Rho modulators, phosphatases governing focal adhesion dynamics, and transcriptional nodes connected to epithelial–mesenchymal transition (EMT).^5,7,8^ Functional perturbations of miR-31 often produce stronger effects on 3D invasion, which requires ECM engagement, proteolysis, and traction generation, than on 2D migration on rigid substrates. This divergence reflects the biology miR-31 touches, integrin activation cycles, invadopodia maturation, and MMP-2/-9 activity are more critical in 3D contexts. Notably, miR-31′s role can be context-dependent across tissues; therefore, readouts that directly couple target engagement to phenotype are vital for mechanistic inference in each model.^5,8,12^

Antisense oligonucleotides (ASOs), including phosphorothioate-backbone, 2′-OMe/2′-F, and locked nucleic acid (LNA) chemistries, remain the dominant modality for miRNA inhibition.^13,14^ Several have reached the clinic in nononcology settings, validating the concept.^15^ However, oncology presents tougher delivery barriers: (i) nuclease degradation and protein binding in serum; (ii) rapid renal clearance for small constructs; (iii) hepatic/splenic uptake and off-target sequestration; (iv) tumor penetration in heterogeneous vasculature; (v) inefficient cellular uptake; and (vi) endosomal escape, frequently the bottleneck for cytosolic access. Chemical stabilization improves exposure but can exacerbate protein binding and reduce endosomal release. Lipid nanoparticles (LNPs) and polymeric carriers enhance uptake but add complexity and sometimes toxicity. A recurring translational lesson is that intracellular bioavailability, not merely circulation half-life, predicts functional potency.

Gold nanoparticles (AuNPs) offer an attractive balance of surface chemistry control, optical readouts, and manufacturability.^15,16^ Dense ligand shells formed via Au–S chemistry enable high oligonucleotide loading and mixed-monolayer engineering (e.g., PEG for stealth, peptides/sugars for targeting, ionizable or pH-responsive moieties for trafficking control). Optical plasmon resonances provide nondestructive quality control and, if desired, photonic actuation.^15,16^ Importantly, AuNPs support ratiometric surface design, as functional groups can be precisely titrated to interrogate how charge density, hydrophilicity, and ligand topology shape uptake routes (clathrin-mediated endocytosis vs macropinocytosis), intracellular sorting, and protein corona formation. These features, coupled with scalable wet-chemistry synthesis and batch-to-batch optical QC, make AuNPs competitive scaffolds for RNA delivery.^16–19^

Molecular beacons (MBs) are stem-loop oligonucleotides labeled with a fluorophore and quencher that fluoresce upon hybridization with a complementary target.^20^ Immobilizing MBs on AuNPs yields nanobeacons, constructs that are physically robust, carry high local oligo density, and remain quenched until target binding partially liberates the dye from the quenching environment. For miRNAs, this architecture is especially powerful as functional knockdown (via antisense complementarity) can be directly coupled to a hybridization readout, enabling spatial validation of target engagement.^21^ Readout-enabled systems are poised to resolve long-standing ambiguities in miRNA therapeutics, namely, whether weak phenotypes arise from delivery failure, endosomal trapping, or true biology.

Most nanoparticles of ∼ 80–120 nm hydrodynamic diameter are avidly internalized yet traffic to late endosomes and lysosomes. Numerous escape tactics have been developed: (i) proton-sponge polymers and dendrimers; (ii) pH-responsive amphiphiles that become membrane-active in acidic compartments; (iii) fusogenic peptides derived from viral proteins or histidine-rich sequences; (iv) ionizable lipids grafted into mixed monolayers; and (v) photochemical internalization, using photosensitizers to locally disrupt endosomal membranes. Each approach trades efficiency against cytotoxicity, immunogenicity, and formulation complexity.^22,23^ Critically, few platforms can quantify endosomal escape in the same particles used for therapy. Nanobeacons with fluorescent readouts combined with reporters like Gal8-GFP or split-luciferase assays provide a rational way to compare escape chemistries under identical delivery conditions.^24,25^

Despite advances, three gaps remain: (1) a lack of mechanism-anchored phenotyping that cleanly separates 3D invasion from 2D migration and cytotoxicity; (2) limited integration of miRNA kinetics (functional sequestration versus delayed abundance changes) with transcriptome remodeling; and (3) underuse of histology-only *in vivo* end points to demonstrate local biological effects without overinterpreting size dynamics. Platforms that combine target-engaged delivery with multiplex readouts across these axes are needed to progress from proof-of-concept to design rules.

We address these gaps by engineering PEGylated Au nanobeacons bearing a Cy3-labeled anti-miR-31-5p hairpin and by evaluating them across a mechanistically aligned panel: (i) physicochemical control (UV–vis plasmonics, DLS/PDI, ζ-potential); (ii) cellular trafficking (confocal colocalization with lysosomal tracers and TEM); (iii) function-first phenotyping that contrasts migration versus invasion in A549-Luc (high miR-31) and RERF-LC-Sq1 CMV-Luc (low miR-31) cells while monitoring viability; (iv) molecular kinetics (time-course miR-31 qRT-PCR) and transcriptomics to map early network shifts and (v) histology-only *in vivo* analysis (H&E, DAPI/Cy3, *K*_i_-67) to spatially validate intratumoral presence and microenvironmental remodeling. Altogether, we aim to establish a mechanistic through-line from nanoparticle chemistry to invasion control and to provide concrete design levers, particularly around endosomal escape, that will inform next-generation, readout-enabled miRNA nanotherapies.

## Experimental Section

### Reagents

Citrate-capped Au nanoparticles (AuNPs; 60 nm core, Sigma-Aldrich #742015), thiolated PEG (5 kDa, Sigma-Aldrich #11124), thiolated DNA/RNA hybrid molecular beacons (anti-miR-31-5p Cy3-labeled; custom, Merck/Eurogentec), dimethyl sulfoxide (DMSO; Sigma-Aldrich #D8418), dithiothreitol (DTT, Sigma-Aldrich #D0632), ethyl acetate (Sigma #319902), NAP-5 columns (Sigma #GE17-0853-02), phosphate buffer (10 mM, pH 8), sodium sodecyl sulfate (SDS, Sigma-Aldrich #L3771), sodium chloride (NaCl, Sigma-Aldrich #S7653), ProLong Glass Antifade (Invitrogen #P36980), 4′,6-diamidino-2-phenylindole (DAPI, Invitrogen #D1306), fluorescein isothiocyanate (FITC)-dextran 10 kDa (Sigma #FD10S), paraformaldehyde (PFA; Sigma #P6148), osmium tetroxide, potassium ferrocyanide, tannic acid, uranyl acetate, Reynolds lead citrate (Electron Microscopy Sciences), Zombie Violet (BioLegend #423114), TaqMan Fast Advanced Master Mix (ThermoFisher #4444964), TaqMan Advanced miRNA Assays (hsa-miR-31 and RNU6B), High-Capacity cDNA Reverse Transcription Kit (ThermoFisher #4374966), hydroxyethyl-cellulose (HEC, Natrosol 250 HX pharm, Ashland), anti-*K*_i_-67 (Bioworld #BS9931M), EnVision+ HRP Rabbit (Agilent Dako #K4003), ultrapure DNase/RNase-free distilled water (Thermofisher Scientific #10977049), sodium phosphotungstic acid (Sigma #P4006), phosphate buffered saline (PBS; Sigma-Aldrich #P4417-100TAB).

### Cell Culture

A549-Luc, RERF-LC-Sq1 CMV-Luc and WI-38 VA13 Sub2RA cells were acquired from the Japanese Collection of Research Bioresources (JCRB) cell bank. A549-Luc cells (JCRB1414, lot 11212019) were cultured in Dulbecco’s Modified Eagle Medium (DMEM; Gibco #10313021) supplemented with 2 mM Glutamax (Gibco #35050061), 1% nonessential amino acids (NEAA; Gibco #11140050), 1% penicillin/streptomycin (Pen/Strep; Gibco #15140122) and (10% FBS). RERF-LC-Sq1 CMV-Luc cells (JCRB1691, lot 08242016) were cultured in Roswell Park Memorial Institute (RPMI; Gibco #21870076) medium, supplemented with 2 mM Glutamax, 1% Pen/Strep and 10% FBS. WI-38 VA13 Sub2RA (JCRB9057, lot 06132014) were cultured in DMEM supplemented with 2 mM Glutamax, 1% NEAA, 1% sodium pyruvate (Gibco #11360070), 1% Pen/Strep and 10% FBS. A549-Luc and RERF LC Sq1 CMV-Luc both stably express the firefly luciferase enzyme. For pretreatments, cells received AuNPs@PEG or AuNPs@PEG@α-miR-31-5p at 0.625–20 nM for 24–72 h (internalization/viability) or 10–20 nM for 48–120 h (migration/invasion, wound healing) as indicated below.

### Identification of miRNA Targets: miRNA Arrays, Target Prediction and Gene Ontology Analysis

To obtain an initial characterization of the lung cell lines in terms of their miRNA expression, the cells were cultured as previously described in 6-well plates, in three independent biological experiments. At least 1 × 10^6^ cells were collected, washed with PBS and pelleted by centrifugation at 2500*g*. The total RNA was extracted as described below and sent to an external laboratory for sequencing (ATLAS Biolabs GmbH). A549-Luc and RERF-LC-Sq1 CMV-Luc analyses were performed using the lung fibroblast cell line WI-38 VA13 Sub2RA as the control group. The top 100 significantly differentially expressed miRNAs were obtained and used for further analysis.

To identify miRNA targets, the results of the miRNA arrays were analyzed considering a fold-change cutoff of 2 and a significance level of 5%. Up- and downregulated miRNAs were identified, as well as common or exclusive miRNAs expressed on each cell line. The visualization of the common or exclusive miRNAs for each cell line was obtained using Venny 2.1.0.^26^ Other visualization graphs of the top 100 miRNAs were obtained using the Thermo Fisher Scientific/Affymetrix Transcriptome Analysis Console software.

Once the target-miRNA was identified (hsa-miR-31), a target prediction database, TargetScan (release 8.0, September 2021, https://www.targetscan.org/vert_80/),^27^ was used to obtain the list of genes it regulates. From this list, the genes showing a cumulative weighted context + + score (CWCS) of ←0.4 were selected. Duplicates were removed when detected and the final list of genes was used for pathway and Gene Ontology analyses, through the Database for Annotation, Visualization and Integrated Discovery (DAVID, https://davidbioinformatics.nih.gov/).^28,29^ The GOTERM_BP_FAT parameter was used to analyze the ‘Biological process’ term. The resulting table was extracted, and the top 100 significant results related were used. These results were classified in more general biological processes using the Gene Ontology Browser (https://www.informatics.jax.org/vocab/gene_ontology) by searching for the Go Terms and grouping the results in the main Biological Processes so that a simpler graph could be obtained.

### Nanoconjugate Construction

#### Synthesis of PEGylated AuNPs

Commercial citrate-capped AuNPs (60 nm core) were PEGylated using thiolated PEG (5 kDa). PEG stock (200 mg/mL in DMSO) was diluted to 1 mg/mL in nuclease-free water and reacted with AuNPs to displace citrate via Au–S chemistry, yielding AuNPs@PEG.

#### Construction of Au Nanobeacons (AuNPs@PEG@α-miR-31-5p)

Lyophilized, thiolated Cy3-beacons (Supporting Table S1) were reconstituted in nuclease-free water, reduced with DTT (0.1 M, 2 h, RT, 30 rpm, protected from light), extracted three times with ethyl acetate, and purified on NAP-5 columns (10 mM phosphate, pH 8). Purified oligos were quantified by UV–vis (Nanodrop). Beacons were mixed with AuNPs@PEG at 1:100 (AuNP:oligo) in phosphate buffer with 0.01% w/v SDS (AGE-I), incubated 20 min (15 rpm), and salt-aged stepwise by adding AGE-II (0.01% w/v SDS, 1.5 M NaCl in 10 mM phosphate, pH 8) to reach final NaCl concentrations of 0.05, 0.1, 0.2, and 0.3 M (20 min between steps, RT). Suspensions were then mixed for 16 h (15 rpm, RT, dark) and centrifuged (60 nm cores: 20,000*g*, 20 min). Pellets were resuspended in nuclease-free water and centrifugation steps were repeated in a total of three times; supernatants were retained for loading analysis.

#### Beacon Loading/Functionalization Efficiency

Free Cy3-beacon in supernatants was quantified by fluorescence (λ_em_ = 570 nm) against a Cy3 calibration curve to compute oligo:Au ratios and % functionalization per batch.

### Physicochemical Characterization

#### UV–vis Plasmonics

Spectra (400–900 nm) were acquired at 1 nM in quartz cuvettes on a Shimadzu UV-2401 PC, using the UV-Probe 2.43 software. Surface plasmon resonance (SPR) peaks were recorded and particle concentration estimated by Beer–Lambert using ε for 60 nm AuNPs (1.73 × 10^10^ M^–1^ cm^–1^).

#### Dynamic Light Scattering (DLS) and ζ-Potential

Samples (1 nM) were measured on an Anton Paar Litesizer 500, using the Kalliope software. Hydrodynamic diameter (HD) and polydispersity index (PDI) were recorded from triplicate size series (1 mL, quartz cuvette). ζ-Potential was measured in Omega cuvettes (triplicate zeta potential series).

#### TEM of Formulations (Negative Stain)

One drop of each sample was deposited on carbon-coated copper grids (EM-Tec, 200 mesh), air-dried, stained with 2% (w/v) sodium phosphotungstic acid (pH 7, 40 s), blotted, and imaged (Hitachi 8100). Particle diameters (*n* = 50 per sample) were quantified in ImageJ.

#### Stability Assays

For select conditions (time, protein exposure), 1 nM nanobeacons were incubated in nuclease-free water or in PBS 1× with 10% fetal bovine serum (FBS; Gibco #10270106), at 4 °C, RT and 37 °C. The hydrodynamic diameter and polydispersity index were evaluated using DLS at days 7, 14, 21, 30 and 60.

### Cellular Internalization and Lysosomal Colocalization

#### Flow Cytometry (Cy3 Readout)

A549-Luc and RERF-LC-Sq1 CMV-Luc (100,000 cells/well, 24-well plates) were treated with 0.625–20 nM nanobeacons for 24, 48, or 72 h. Cells were trypsinized, washed, fixed in 4% PFA (30 min, RT, dark), washed, resuspended in PBS and analyzed on BD FACS Aria III (≥10,000 single cells). MFI (Cy3) and %Cy3^+^ cells were computed in FlowJo v10.

#### Widefield Microscopy

For time-course uptake, cells on coverslips (100,000 cells/well, 24-well plates) were treated with 10 or 20 nM for 24/48/72 h, fixed (4% PFA), counterstained with DAPI, mounted on glass slides in a drop of ProLong Glass Antifade, and imaged on a Zeiss Z2 microscope, using the Zen Blue software.

#### Lysosomal Colocalization

A549-Luc were plated (100,000 cells/well, 24-well plates) on coverslips, incubated with FITC-dextran (100 μg/mL, 24 h), washed, and then treated with 10 nM nanobeacons for 72 h, fixed (4% PFA), stained with DAPI, mounted on glass slides in a drop of ProLong Glass Antifade, and imaged on a LSM710 confocal microscope (Zeiss). Z-stacks were collected using a 63× magnification objective lens with immersion oil; 128 slices; 0.32 μm step; *z*-depth 40.64 μm; scan zoom 1.5. ImageJ was used to compute average-intensity projections and channel merges.

#### TEM of Treated Cells

A459-Luc cells on coverslips were treated (10 nM; 30 min, 72 h), fixed (2% PFA/2% glutaraldehyde in PBS), postfixed (1% OsO_4_ + 1.5% K_4_[Fe­(CN)_6_] on ice), tannic acid (1%, 30 min), ethanol-dehydrated (50–100%) and embedded in Epon (EMbed-812). Coverslips were removed in liquid nitrogen, sectioned (60–70 nm; Reichert Ultracut S), poststained (uranyl acetate, lead citrate), and imaged on a Hitachi H-7650 microscope (AMT XR41-M camera). Processing and imaging were performed as a core service.

### Viability by Flow Cytometry

A549-Luc and RERF-LC-Sq1 CMV-Luc (100,000 cells/well, 24-well plates) were treated (0.625–20 nM; 24/48/72 h), detached with trypsin-EDTA (Gibco #25200072), stained with Zombie Violet (1:500 in PBS 1×, 15 min, RT, dark), washed, fixed in 4% PFA and analyzed on a FACS Aria III cytometer. Data was processed in FlowJo v10; ≥ 10,000 single cells were collected per sample.

### Migration and Invasion Assays

#### Scratch (Wound-Healing)

A549-Luc (100,000 cells/well, 24-well plates) were left to attach for 24 h after plating, and treated with nanobeacons (10 or 20 nM; 48 or 72 h). The cell monolayer was scratched with a P200 tip, rinsed, and imaged at 0–120 h (24 h intervals) on a Zeiss Axiovert 40 microscope. The wound area was quantified in ImageJ.

#### Transwell Migration/Invasion

A549-Luc (10,000 cells/well, 96-well plates) were left to attach for 24 h after plating, and treated with nanobeacons (10 or 20 nM; 72 or 120 h). Cells were detached, washed, and resuspended in medium containing 2% FBS (500 μL). Migration: cells were added to the upper chamber of 8-μm pore inserts (Corning #353097) in 24-well plates, containing medium with 10% FBS in the lower chamber (500 μL), and incubated for 24 h (37 °C, 5% CO_2_). Invasion: Matrigel-coated inserts (Corning #354480) were rehydrated (serum-free medium) and used similarly (24 h invasion). Membranes were ethanol-fixed (70% v/v), upper-surface cells were removed with a cotton swab and lower-surface cells were DAPI-stained (5 μg/mL, 5 min). The membranes were mounted on a glass slide containing a drop of ProLong Glass Antifade. The nuclei were imaged on a Zeiss Z2 microscope (10 fields/insert, 10×) and counted in ImageJ.

### miR-31 Quantification by qRT-PCR

A549-Luc and RERF-LC-Sq1 CMV-Luc (50,000 cells/well, 48-well plates) were left to attach for 24 h after plating, and treated with nanobeacons (10 or 20 nM) for 24, 48, 72, 96, 120, and 144 h. Total RNA was extracted following the manufacturer’s instructions, and 1 μg RNA/sample was reverse-transcribed with the High-Capacity kit in a 35-μL reaction modified to include both miR-31 and RNU6B RT primers (per-reaction master-mix volumes in Supporting Table S5); GeneAmp PCR System 9700 conditions: 25 °C 10 min, 37 °C 120 min, 85 °C 5 min, 4 °C hold. qPCR was run on QuantStudio 5 with TaqMan Fast Advanced Master Mix using miR-31 and RNU6B assays (20×) (per-reaction master-mix volumes in Supporting Table S6). Cycling: 95 °C 20 s, then 40 cycles of 95 °C 1 s/60 °C 20 s. Data were analyzed by the 2^–ΔΔCt^ method with RNU6B as endogenous control.

### RNA-seq

A549-Luc cells (250,000 cells/well, 6-well plates) were left to attach for 24 h after plating and treated with 20 nM nanobeacons for 72 h. RNA was extracted as mentioned and submitted to Novogene for library prep and sequencing. Differential expression was computed (AuNPs@PEG control vs nanobeacon) with Novogene’s NovoMagic pipeline; only *p* < 0.05 genes were considered. Gene Ontology enrichment followed the DAVID-based workflow, as previously mentioned.

### *In Vivo* Study and Histology

Subcutaneous A549-Luc xenografts were established in athymic Balb/C female nude mice (Charles River Laboratories, France) by subcutaneous injection on the right flank (2.5 × 10^6^ cells/mouse) and allowed to grow. Mice were maintained in aseptic conditions with a 12-h light/dark cycle and with standard pellet food and water provided *ad libitum*. When tumors averaged ∼60 mm^3^, mice were randomized into two groups (*n* = 5/group). Mice received intratumoral injections (100 μL) of HEC@AuNPs@PEG (control) or HEC@AuNPs@PEG@α-miR-31-5p (treatment) under isoflurane anesthesia. Postinjection fluorescence imaging (λ_ex_ = 540 nm; Newton FT-500, Vilber) and bioluminescence (IVISbrite D-luciferin, 15 mg/mL in PBS, 200 μL subcutaneous, 5 min uptake) were performed repeatedly over 21 days. At the end point, animals were euthanized (isoflurane overdose, cervical dislocation); tumors and organs were collected, imaged, and fixed in 10% (w/v) formalin in PBS 1×. The organs’ and tumors’ bioluminescence signal was analyzed and quantified using Kuant (Vilber Lourmat), and results were plotted into a heatmap using the GraphPad Prim (version 8.0.2) software. Paraffin sections of the tumors (5 μm) were stained with hematoxylin and eosin (H&E) and for *K*_i_-67. For immunofluorescence (IF), deparaffinized sections were permeabilized (0.3% v/v Triton X-100), DAPI-stained (5 μg/mL), mounted in ProLong Glass Antifade, and imaged on a Zeiss Z2 microscope. Sectioning and immunohistochemistry (IHC) processing were performed by the Nova Medical School’s Histology Facility. The HEC hydrogel was selected to enhance intratumoral retention and limit rapid dispersion; animal weight and welfare were monitored throughout.

*K*_i_-67 IHC was quantified on digitized bright-field micrographs for each tumor (control and treated). Images were analyzed in Python (scikit-image) after color deconvolution using the Ruifrok-Johnston HED matrix to separate hematoxylin and DAB channels. A tumor ROI was first drawn manually to exclude surrounding normal tissue and obvious artifacts. Within this ROI, nuclei were segmented on the hematoxylin channel by adaptive thresholding and morphological cleanup, and DAB-positive nuclei were identified by thresholding the DAB channel and intersecting it with the nuclear mask. The *K*_i_-67 labeling index (LI) was calculated for each tumor as LI (%) = 100 × (number of *K*_i_-67-positive nuclei/total number of nuclei).

Necrosis (IHC, H&E) was quantified using digitized sections that were color-deconvolved (Ruifrok-Johnston HED) to separate hematoxylin and eosin, with each channel normalized to the first-99th percentiles. A manual tumor ROI was drawn to exclude nontumor tissue and artifacts, and a tissue mask removed background. Necrosis was defined adaptively within each tumor as pixels simultaneously low in hematoxylin and eosin (both below the tumor-specific 20th percentile), followed by morphological cleanup (removed small objects/holes ∼200 px and a light closing 2–3 px). Necrotic burden was reported as Necrosis (%) = 100 × necrotic area/tumor ROI area.

Inflammation (peritumoral index) was quantified from the same ROI; a peritumoral ring was generated by dilating the tumor mask (disk ≈20 px; fallback ≈10 px when needed) and subtracting the tumor, intersecting with the tissue mask and excluding necrosis plus a 3–5 px margin. Nuclei were segmented on the hematoxylin channel using median filtering, Otsu/adaptive thresholding and cleanup (remove <12 px), then inflammatory cells were approximated as small, round nuclei within the ring (area 15–200 px; eccentricity ≤ 0.9). The metric was expressed as Inflammation index = number of inflammatory nuclei per 10^5^ pixels of ring area, which was used directly for visualization and statistics.

### Statistics

Unless stated otherwise, experiments were performed in ≥3 independent biological replicates. Results are presented as mean ± SD. Statistical tests were performed using the GraphPad Prim (version 8.0.2) software. Figure-specific statistical tests and n are reported alongside the corresponding data in the manuscript.

## Results and Discussion

### Differential miRNA Profiling Identifies miR-31–5p as a Target for Lung Cancer

We used miRNA arrays to evaluate the expression of 6634 miRNAs on A549-Luc and RERF-LC-Sq1 CMV-Luc cells, relatively to control cells (WI-38 VA13 Sub2RA). Of these, the top 100 differentially expressed miRNAs were listed (Supporting Information, Table S2) and analyzed (Figure 1A). Thirteen miRNAs are exclusively expressed on A549-Luc cells, and 25 miRNAs are exclusively expressed on RERF-LC-Sq1 CMV-Luc cells, and the two cell lines share the expression of 62 miRNAs (Figure 1B). According to these results, we selected hsa-miR-31-5p as the top candidate to be used as target for the development of anti-miRNA nanobeacons. As shown in Figure 1C, this miRNA is much more expressed in A549-Luc cells than in the RERF-LC-Sq1 CMV-Luc cells (log_2_ signal values = 11.88 vs 0.39, respectively). Also, the volcano plot in Figure 1D highlights miR-31-5p as a great target as it shows the highest fold-change and the most significant p-value obtained (3.98 × 10^–16^) when comparing to the other differentially expressed miRNAs. These results emphasize this miRNA as a key player in mRNA and gene regulation, and especially, show a possible reason for the observation of different biological roles between the two cell lines. Moreover, several studies have associated miR-31 with an oncogenic role in lung cancer. In fact, other authors reported that the increased expression of miR-31 in patient tumors correlated with adverse outcomes, and that it was associated with tumor growth, increased EMT, lymph node metastases, and lower patient survival.^9–11,30–32^ Gene Ontology analysis shows that miR-31-5p regulates 1325 genes, mostly involved in cell motility, cell communication, metabolic processes and response to different stimuli (Figure 1E). These results helped us to understand the functional effects that should be evaluated in lung cancer models, since the conjugation of anti-miR-31-5p molecular beacons with gold nanoparticles for miR-31-5p silencing has the possibility to revert tumor-associated processes, such as increased migration, invasion and proliferation of tumor cells.

**Figure 1:**
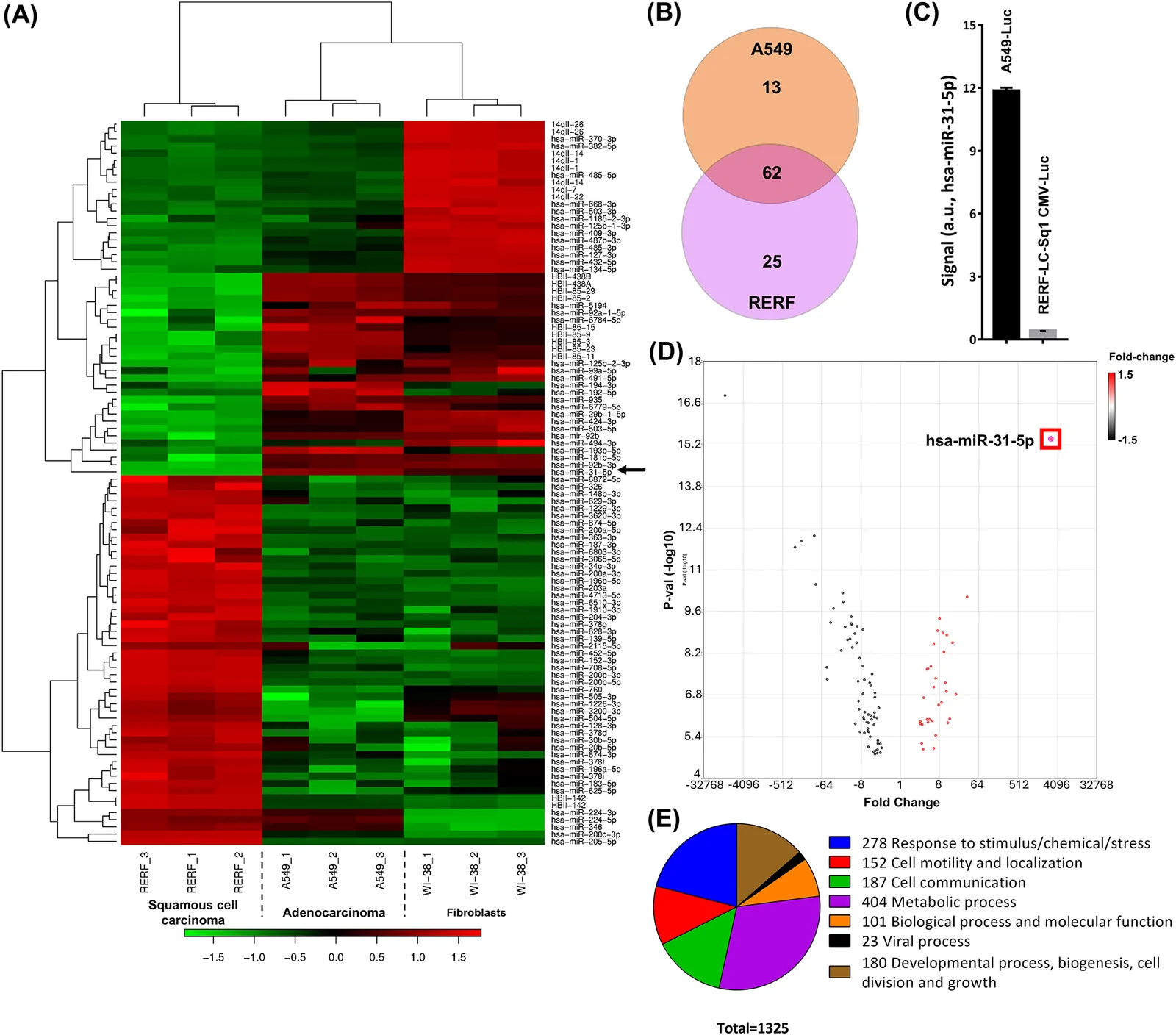
Selection of miR-31-5p as the target miRNA. (A) miRNA arrays were performed to evaluate miRNA expression on two lung cancer cell lines (A549-Luc, adenocarcinoma; RERF-LC-SQ1 CMV-Luc, squamous cell carcinoma). Expression was analyzed relatively to the expression in lung fibroblasts (WI-38 VA13 Sub2RA) and the top 100 significantly expressed miRNAs for each cell line were selected for analysis. The black arrow indicates hsa-miR-31-5p. (B) Venn diagram showing the distribution of the top 100 miRNAs on both lung cancer cell lines. 62 miRNAs are common to both cell lines, while A549-Luc shows 13 uniquely expressed miRNAs and RERF-LC-SQ1 CMV-Luc shows 25 uniquely expressed miRNAs. (C) Bar graph showing the expression of hsa-miR-31-5p on A549-Luc cells (black) and on RERF-LC-SQ1 CMV-Luc cells (gray). Hsa-miR-31 5p is downregulated exclusively in RERF-LC-SQ1 CMV-Luc cells, being upregulated in A549-Luc cells. (D) Volcano plot highlighting the hsa-miR-31 5p miRNA among the top 100 differentially expressed miRNAs. Downregulated miRNAs are represented in black, on the left side of the plot, while upregulated miRNAs are represented in red, on the right side of the plot. (E) Gene-ontology analysis for miR-31-5p highlighting pathways associated with metabolic processes, response to stimuli, cell communication, motility and locomotion and developmental processes.

### Construction and Physicochemical Profiling of Au Nanobeacons

We built the nanobeacon in two modular steps: (i) PEGylation of citrate-capped ∼ 60 nm Au cores (Supporting Figure S1A–E) and (ii) covalent grafting of a Cy3-labeled α-miR-31-5p hairpin (molecular beacon) via Au-thiol chemistry under salt-aging.^21^ Briefly, thiolated hairpins were reduced with DTT, purified (organic extraction + NAP-5 column), and combined with PEG-AuNPs at a 1:100 AuNP:oligo ratio. Ionic strength was increased stepwise (0.05 → 0.1 → 0.2 → 0.3 M NaCl in 0.01% SDS, 10 mM phosphate buffer, pH 8) with gentle mixing, followed by overnight incubation and triple centrifugation to remove unbound oligos; supernatants were retained to quantify loading. This workflow preserves colloidal stability while driving dense, covalent monolayer formation (Scheme 1B). Cy3 was selected for the 60 nm platform because its emission (∼570 nm) overlaps the ∼540 nm plasmon band, maximizing quenching in the closed hairpin (“off”) and fluorescence recovery upon hybridization (“on”) (Scheme 1C). Supernatant analysis against a Cy3 calibration curve yielded a mean functionalization efficiency of 73.9 ± 37.4% for the α-miR-31-5p beacon (batch variability noted), whereas the α-miR-31-3p conjugation failed (0% efficiency) (Supporting Figure S1F–K), and those particles were not advanced in cellular assays, despite being further characterized.

The UV–vis spectra captures the essential quality controls, across the three productive steps (AuNPs, AuNPs@PEG, AuNPs@PEG@α-miR-31-5p), the SPR band remains centered near ∼ 540 nm (Figure 2A), and the extracted peak positions cluster tightly with only subnanometer drift (Figure 2B, Table 1). This invariance is important mechanistically, since large red-shifts or band broadening would imply interparticle coupling/aggregation or a drastic refractive-index perturbation of the near field, either of which would compromise delivery. Instead, the constancy of both position and line shape indicates that the ligand additions are in the “thin shell” regime, modifying the local dielectric environment without inducing plasmonic coupling. By contrast, the α-miR-31-3p attempt shows a small blue-shift and broader profile (Figure 2A–B), a signature consistent with inefficient coupling and partial dispersion during salt-aging; it functions as an internal negative control that highlights how failed conjugation perturbs the optical readout.

**Figure 2:**
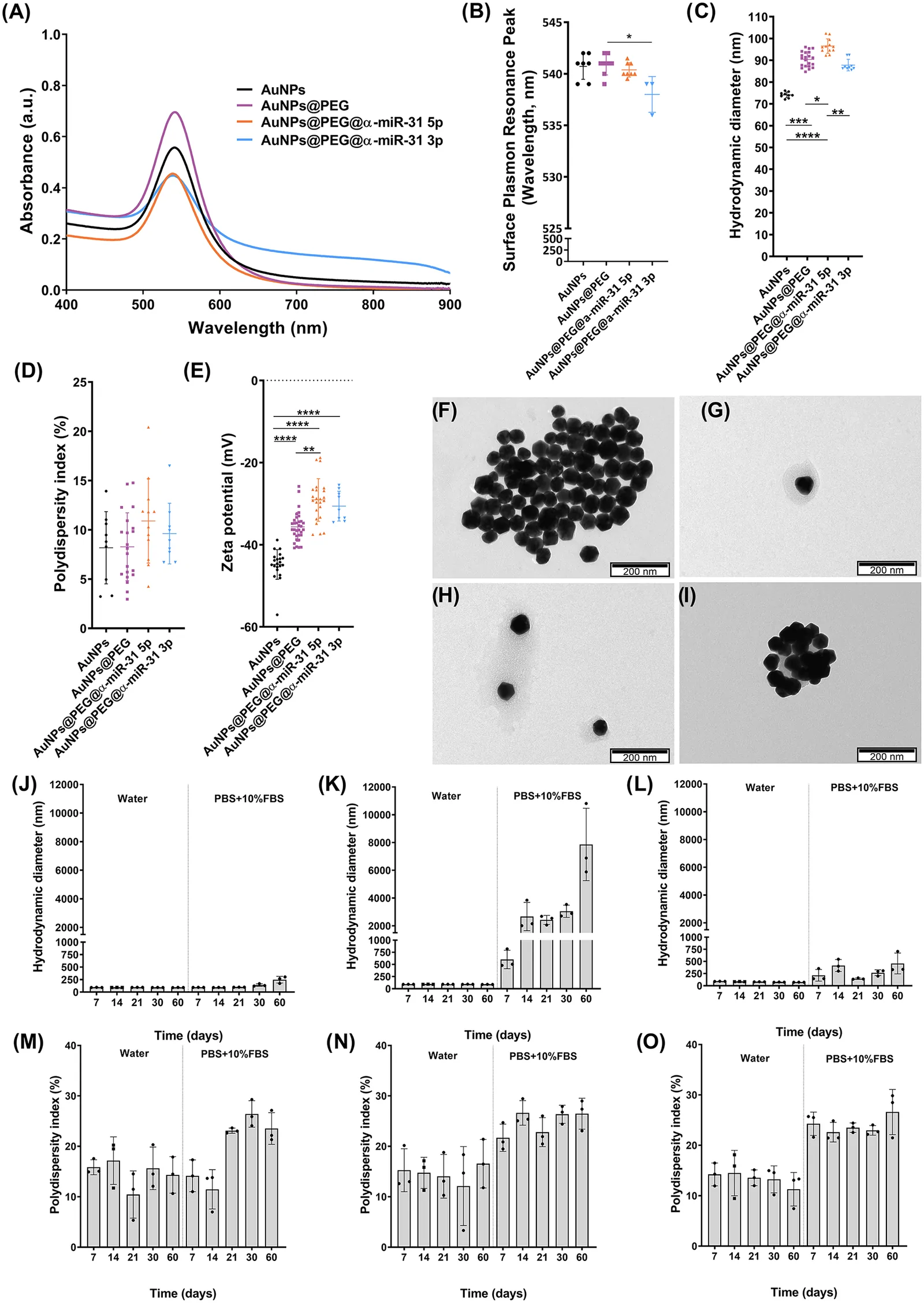
Construction and physicochemical profiling of miR-31 nanobeacons. (A) UV–vis spectra show a stable surface plasmon resonance (SPR) near ∼ 540 nm across all formulation steps (AuNPs → AuNPs@PEG → AuNPs@PEG@α-miR-31-5p); the α-miR-31-3p attempt displays a slight blue shift and line-shape change consistent with poor conjugation. (B) Extracted SPR maxima (nm) confirm minimal drift for AuNPs, PEGylated cores, and 5p nanobeacons. (C) Dynamic light scattering (DLS) reports the expected layer-by-layer increase in hydrodynamic diameter (HD) from core to PEG to 5p; the 3p sample tracks closer to PEG-only. (D) Polydispersity indices (PDI) remain low for all productive steps, supporting monodisperse suspensions. (E) ζ-Potential shifts from strongly negative (citrate) to moderately negative values after PEG and beacon grafting, consistent with citrate displacement and a brush-stabilized organic shell; significance markers reflect multiple-comparison tests (*p < 0.05; **p < 0.01; ***p < 0.001; ****p < 0.0001). (F–I) TEM micrographs show compact ∼60 nm cores; a faint negative-stain corona is evident for PEG and 5p nanobeacons, whereas the 3p sample lacks a clear halo (scale bars: 200 nm). (J–L) Longitudinal HD in water (left panels) remains stable over 60 days; increases in PBS + 10% FBS (right panels) reflecting protein-corona formation rather than catastrophic aggregation. (M–O) Corresponding PDIs over time mirror this trend, remaining within a manageable range for all constructs. Data sets show incubation at 4 °C (J/M), room temperature (K/N) and 37 °C (L/O). Data are represented as the mean ± SD; *n* values are indicated on the plots. Together, the unchanged SPR, stepwise HD growth with low PDI, moderated ζ-potential, intact core morphology with an organic shell, and predictable behavior in protein-rich media verify successful assembly and serum tolerance of AuNPs@PEG@α-miR-31-5p.

**Table 1:** Characterization of 60 nm-core AuNPs, AuNPs@PEG and Au Nanobeacons^a^

	**AuNPs**	**AuNPs@PEG**	**AuNPs@PEG@α-miR-31-5p**	**AuNPs@PEG@α-miR-31-3p**
**core size (nm)**	65.33 ± 11.56	64.42 ± 7.54	66.36 ± 7.97	59.71 ± 7.59
**hydrodynamic diameter (nm)**	74.13 ± 1.31	90.42 ± 3.49	96.53 ± 3.30	87.83 ± 2.66
**polydispersity index (%)**	8.18 ± 3.67	8.28 ± 3.44	10.90 ± 4.29	9.62 ± 3.08
ζ **potential (mV)**	–44.82 ± 3.60	–35.65 ± 3.52	–29.10 ± 5.18	–30.59 ± 3.63
**surface plasmon resonance peak (nm)**	540.7 ± 1.25	540.9 ± 0.99	540.4 ± 0.69	538 ± 1.73

a The values of core size (measured by TEM), hydrodynamic diameter, polydispersity index and ζ-potential (measured by DLS) and surface plasmon resonance (measured by UV–vis spectroscopy) are represented. Values represent the mean ± standard deviation.

Concerning hydrodynamic sizing, this resolves the ligand-layer built with complementary sensitivity. The DLS-derived diameter increases monotonically from the citrate-capped cores to PEG and then to the α-miR-31-5p nanobeacon (Figure 2C), which is precisely the trajectory expected for a compact inorganic core acquiring first a neutral brush (PEG) and then an oligonucleotide shell. Crucially, this growth occurs with low polydispersity (Figure 2D), arguing for a single, well-defined particle population rather than a mixture containing a minority aggregate mode that can bias intensity-weighted DLS. The α-miR-31-3p sample sits near the PEG-only size, reinforcing the interpretation that little to no beacon was successfully grafted. From a delivery perspective, the final hydrodynamic size near ∼100 nm is intentional, once it sits in a well-documented window for efficient endocytosis while avoiding the rapid renal clearance regime (<10 nm) and the splenic filtration issues that accompany larger, polydisperse aggregates.^33,34^ Together with the optical results, the DLS/PDI behavior establishes that any downstream biological effects are unlikely to be artifacts of colloidal instability.

Surface potential trends further to support productive surface chemistry. ζ-potential shifts from strongly negative for citrate-capped AuNPs toward moderately negative values after PEG and beacon grafting (Figure 2E). The citrate displacement and the steric screening provided by a neutral PEG brush, plus a folded hairpin that partly buries polyanionic phosphate backbones, move the ζ-potential into the −30 mV regime. That window is advantageous in serum, sufficiently anionic to limit nonspecific protein adsorption and suppress bridging flocculation, but not so highly charged that ions compress the double layer and promote salt-induced aggregation (a classic DLVO trade-off). It also foreshadows the biological behavior we observe later, in which these moderately negative, brush-stabilized particles tend to accumulate a soft corona without catastrophic growth.

Next, electron microscopy provides orthogonal validation of morphology and shell formation. TEM shows compact, largely spherical cores with a narrow size spread; after functionalization, a faint negative-stain “halo” consistent with an organic shell becomes apparent for the PEGylated particles and the 5p nanobeacon, whereas the 3p sample lacks a clear corona and displays occasional small clusters (Figure 2F–I). The presence of rare clusters without a corresponding plasmonic red-shift indicates these events are not dominant in the bulk and likely arise from grid-drying artifacts or weak secondary interactions rather than formulation failure. The analogy among TEM, UV–vis, and DLS is nontrivial. When all three converge, the probability that hidden, larger modes are dictating the solution behavior is low.

Finally, the longitudinal stability series substantiates that these constructs are not only well built but also fit-for-use under realistic handling conditions. Over 60 days in water, hydrodynamic diameters remain essentially invariant for all formulations (Figure 2J–L, left), and PDIs stay low (Figure 2M–O, left), demonstrating storage resilience. In PBS + 10% FBS, the expected increase in apparent diameter and PDI emerges (Figure 2J–O, right). We interpret this as protein-corona maturation rather than catastrophic aggregation, in which growth proceeds gradually and the distributions remain bounded. Practically, this behavior is desirable for nanomedicine because a reproducible soft corona forms in serum without compromising colloidal integrity, which preserves uptake competence while avoiding embolic risk. Notably, the 5p nanobeacon shows a tempered growth profile compared with inadequately functionalized controls, providing a built-in quality control readout for batch acceptance. Additionally, the 5p nanobeacon showed the capacity to open and close the hairpin (Supporting Figure S2A–B), an essential process to be able to detect and hybridize with the target miRNA, which was accomplished *in vitro* upon incubation with five times more miR-31-5p (Supporting Figure S2C–D). In addition, stability assays in the presence of RNase revealed dose-dependent degradation of the α-miR-31-5p hairpin (Supporting Figure S2E).

Taken together, the unchanged SPR position and line shape (Figure 2A–B), stepwise hydrodynamic growth with low dispersity (Figure 2C–D), moderated ζ-potential consistent with brush stabilization (Figure 2E), intact core morphology with an organic shell (Figure 2F–I), and predictable, protein-tolerant stability over time (Figure 2J–O) converge on a well-constructed, serum-compatible, readout-enabled nanobeacon. The α-miR-31-3p control further underscores the specificity of these signatures by illustrating how failed conjugation perturbs each of the metrics. These materials characteristics are not cosmetic, they are the mechanistic foundation that enables selective anti-invasive activity, benign migration/viability profile, and spatial *in vivo* readouts developed in the remainder of the study.

### Cellular Uptake and Endolysosomal Trafficking

The uptake studies demonstrate that the Cy3-labeled nanobeacons accumulate in cells in a time- and dose-dependent manner and localize primarily to the endolysosomal system. In A549-Luc (miR-31^high^) monolayers, microscopy imaging revealed sparse Cy3 puncta at 24 h that intensified by 48 h and became prominent at 72 h for both 10 and 20 nM, concentrating as perinuclear vesicles; PEG-only controls remained dark, confirming that fluorescence derives from the beacon and not the particle chassis (Figure 3A). RERF-LC-Sq1 CMV-Luc (miR-31^low^) showed the same qualitative trajectory under matched acquisition, albeit with weaker per-pixel signal at early time points (Figure 3B), in line with reduced target copies per cell.

**Figure 3:**
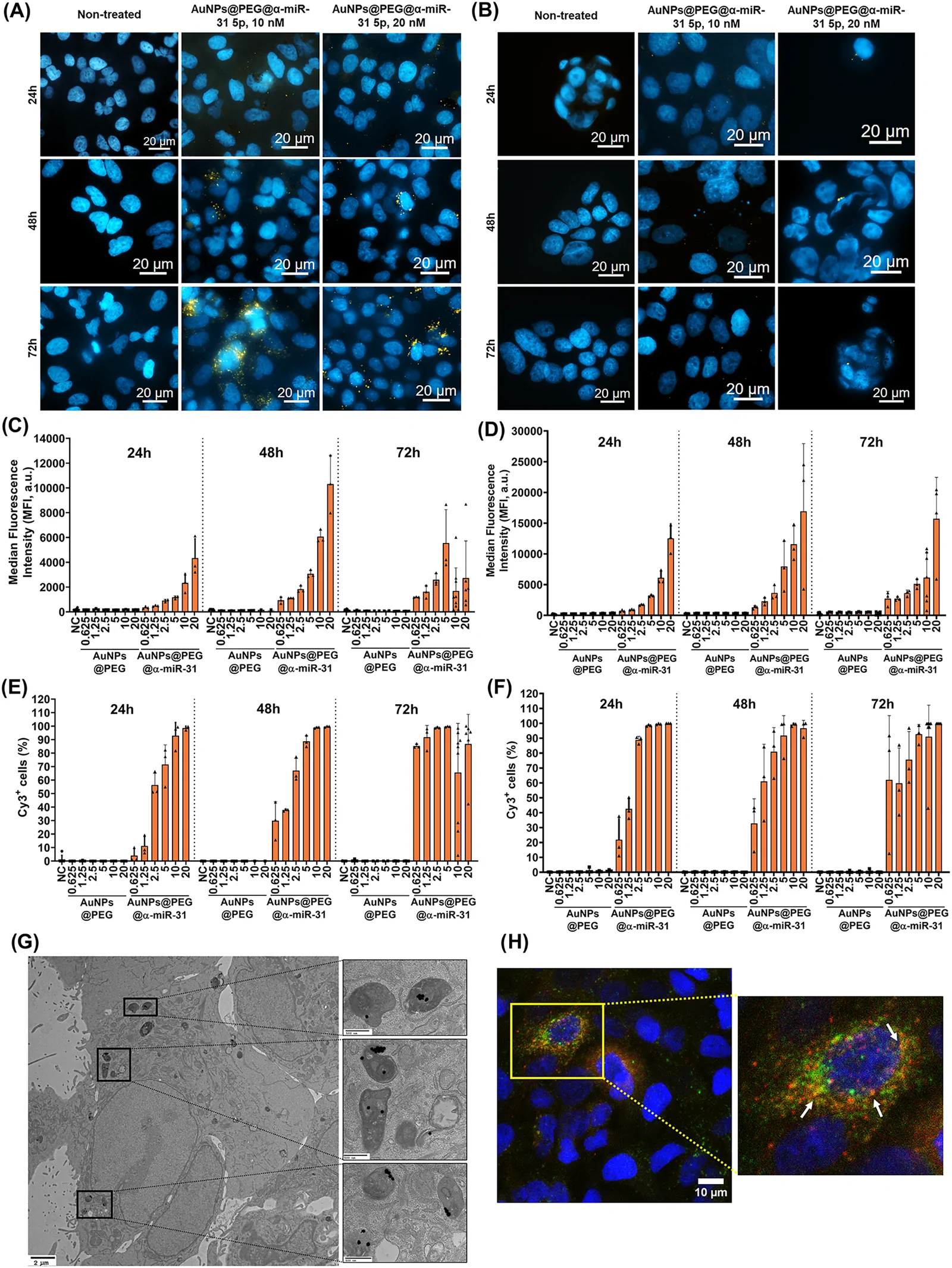
Cellular uptake and endolysosomal trafficking of miR-31 nanobeacons. (A) A549-Luc (miR-31^high^) widefield microscopy images showing time- and dose-dependent Cy3 signal after treatment with AuNPs@PEG@α-miR-31–5p (10 or 20 nM; 24, 48, 72 h). Perinuclear puncta accumulate by 72 h; PEG-only controls are dark. (B) RERF-LC-Sq1 CMV-Luc (miR-31^low^) imaged under identical settings shows the same qualitative kinetics with weaker early signals. DAPI (blue); Cy3 (yellow). Scale bars: 20 μm. (C–D) Flow-cytometry median fluorescence intensity (MFI) for Cy3 in A549-Luc (C) and RERF-LC-Sq1 CMV-Luc (D) at 24/48/72 h across doses, in nM; PEG controls at baseline. (E-F) Corresponding %Cy3^+^ cells for A549-Luc (E) and RERF-LC-Sq1 CMV-Luc (F), showing 24 → 48 h increases and 72 h plateaus with 20 nM > 10 nM; higher late-time readouts in RERF-LC-Sq1 CMV-Luc reflect target-limited beacon activation rather than superior uptake. Bars show mean ± SD (G) TEM of A549-Luc at 72 h after treatment with nanobeacons (10 nM) reveals electron-dense Au cores in membrane-bound vesicles (left, low magnification; right, high-magnification insets) consistent with late endosome/lysosome compartments. Scale bar (left): 2 μm; right insets: 500 nm. (H) Confocal colocalization at 72 h (A549-Luc; 10 nM nanobeacon; 100 μg/mL FITC-dextran) shows substantial Cy3/FITC overlap within vesicles (arrows), confirming endolysosomal routing. Scale bar: 10 μm. Together, imaging and cytometry establish efficient internalization, progressive target-engaged fluorescence, and predominant lysosomal trafficking by 72 h.

Flow cytometry resolves these dynamics quantitatively. In A549-Luc, the Cy3 median fluorescence intensity (MFI) and the fraction of Cy3^+^ cells rose from 24 h → 48 h and then plateaued by 72 h with a clear ordering 20 nM > 10 nM (Figure 3C,E). RERF-LC-Sq1 CMV-Luc exhibited the same kinetics (Figure 3D,F) but, strikingly, the late-time MFI appeared higher than in A549-Luc. This inversion is consistent with target-limited activation, where at low miR-31 levels, a greater fraction of beacons on the nanoparticle can hybridize, using the same intracellular concentration of nanobeacon. This boosts the fluorescence signal without indicating increased uptake. The concordance between imaging and cytometry, together with the uniformly dark PEG controls, argues that the Cy3 signal reflects on-particle hybridization rather than dye desorption or autofluorescence.

Orthogonal subcellular readouts establish the trafficking route. Transmission electron microscopy at 72 h (10 nM) revealed electron-dense Au cores confined within membrane-bound vesicles distributed around the nucleus. Higher-magnification views revealed small intraluminal clusters and morphologies characteristic of late endosomes/lysosomes, compatible with modest corona-mediated clustering during or after uptake (Figure 3G; Supporting Figure S3). Complementarily, confocal colocalization with FITC-dextran, an endolysosomal tracer, demonstrated substantial Cy3/FITC overlap in A549-Luc after 72 h (10 nM nanobeacon; 100 μg/mL dextran; 63× oil, z-stack), confirming lysosomal accumulation under live-cell-relevant conditions (Figure 3H). When considered alongside the hydrodynamic size near ∼100 nm and moderately negative ζ-potential established in Figure 2, these data support classical endocytic entry, most plausibly clathrin-mediated uptake and/or micropinocytosis, followed by lysosomal sorting; rigorous pathway attribution would require pharmacologic or genetic inhibition and is beyond our present scope.

Crucially, lysosomal residence is compatible with the biology observed downstream. miRNA networks are exquisitely sensitive to partial cytosolic access, which means that even a small escape fraction, arising from vesicular leakage or fusion errors, can sequester functional miR-31 and trigger measurable phenotypes without bulk endosomal rupture. Thus, Figure 3 establishes the trafficking context for the platform, efficient internalization, progressive target-engaged fluorescence at 10–20 nM, and predominant lysosomal routing by 72 h, providing the mechanistic bridge between the nanobeacon’s materials properties and its selective anti-invasive action reported later.

### Selective Functional Modulation: Invasion Down, Migration and Viability Unchanged

Pre-exposure of A549-Luc cells to AuNPs@PEG@α-miR-31-5p produced a selective anti-invasive phenotype while leaving planar migration and viability unchanged. In Matrigel-containing transwells, representative DAPI micrographs show visibly fewer nuclei on the underside of the membrane for A549-Luc treated with the nanobeacon versus PEG control, with the effect more pronounced at 20 nM than at 10 nM (Supporting Figure S4A). Quantification confirms a significant reduction in invasion at 72 h, with a clear dose ordering (20 nM > 10 nM; Figure 4A, right vs left). Extending pretreatment to 120 h preserved this inhibition but did not deepen it, consistent with a functional plateau once accessible miR-31 pools are sequestered (Figure 4B; Supporting Figure S4B). In contrast, the low-miR-31 cell line RERF-LC-Sq1 CMV-Luc shows no material change in invasion after nanobeacon exposure (Figure 4C; Supporting Figure S4C), supporting on-target dependence of the phenotype.

**Figure 4:**
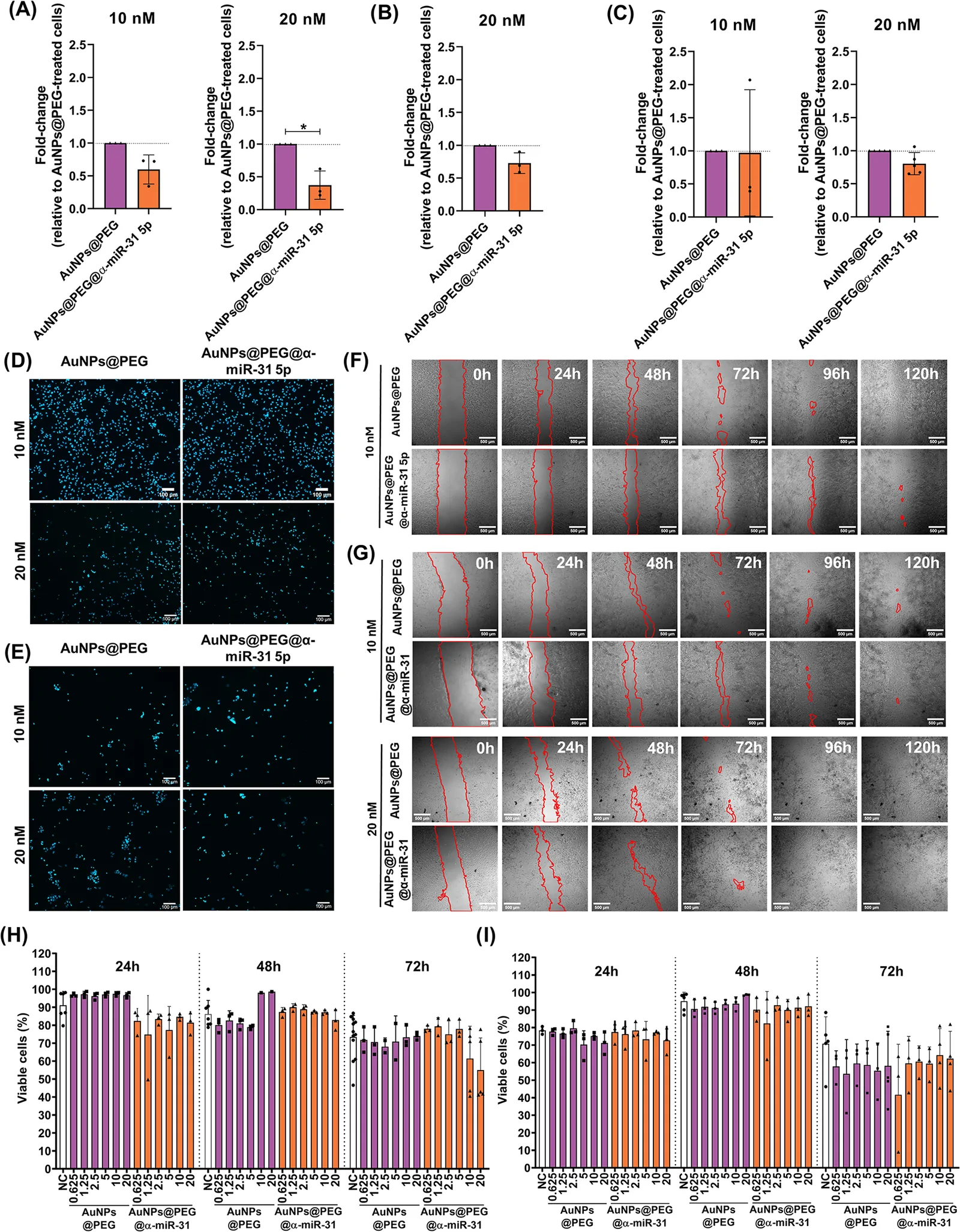
Selective functional modulation: invasion down, migration and viability unchanged. (A–C) Quantified invasion fold-change (relative to PEG control) for: A549-Luc at 10 and 20 nM, after pretreatment for 72 h (A) and after pretreatment for 120 h, at 20 nM (B), and for RERF-LC-Sq1 CMV-Luc, after pretreatment for 72 h at 10 and 20 nM (C); A549-Luc shows significant inhibition with 20 nM > 10 nM, whereas for RERF-LC-Sq1 CMV-Luc remains ∼ 1× (on-target selectivity). (D-E) Representative transwell images (DAPI-stained nuclei on the underside) of A549-Luc (D) and RERF-LC-Sq1 CMV-Luc (E) pretreated for 72 h with AuNPs@PEG (control, left) or AuNPs@PEG@α-miR-31–5p (right) at 10 (top) or 20 nM (bottom). No obvious change is observed upon nanobeacon treatment, indicating preserved 2D motility. Scale bars: 100 μm. (F-G) Scratch (wound-healing) time courses (0–120 h) for A549-Luc after 48 h pretreatment at 10 nM (F) or 72 h pretreatment at 10 and 20 nM (G); red overlays delineate the wound edge; no systematic effect on gap closure is observed versus PEG controls. (H–I) Viability (%) by Zombie dye flow cytometry for A549-Luc (H) and RERF-LC-Sq1 CMV-Luc (I) across 24/48/72 h and indicated doses; values remain high (>∼90%) for PEG and nanobeacon conditions, excluding cytotoxic confounding. Bars show mean ± SD; symbols denote independent replicates; significance markers on bar charts correspond to multiple-comparison tests (*p < 0.05; **p < 0.01; ***p < 0.001; ***p < 0.0001). The coordinated pattern, decreased invasion, unchanged migration and viability, demonstrates selective, ECM-dependent anti-invasion.

Migration readouts corroborate the selectivity. Transwell assays showed no significant differences for both the A549-Luc cells (Figure 4D; Supporting Figure S4D) and the RERF-LC-Sq1 CMV-Luc cells (Figure 4E; Supporting Figure S4E). Scratch assays mirrored this neutrality in A549-Luc, as they revealed no systematic change in gap-closure kinetics across 0–120 h at 10 nM relative to PEG control, after a pretreatment of 48 h (Figure 4F; Supporting Figure S4F). In addition, no difference was observed in the migration of cells relative to PEG control when pretreatment was increased to 72 h, at either 10 or 20 nM. (Figure 4G; Supporting Figure S4G). These results indicate that the nanobeacon does not impair 2D motility on rigid substrates under conditions that blunt 3D invasion through ECM. This divergence – decreased invasion and preserved migration, implies programs specific to matrix engagement and pericellular proteolysis (e.g., invadopodia maturation, integrin activation cycling, MMP activity) rather than generalized deficits in actomyosin-driven movement.

Orthogonal cell-health measurements exclude cytotoxic confounding. Zombie dye flow cytometry shows high viability (>90%) across all doses and time points for both PEG control and nanobeacon in A549-Luc (Figure 4H) and RERF-LC-Sq1 CMV-Luc (Figure 4I) cells. Thus, reduced invasion cannot be attributed to loss of viable cells. Furthermore, cell cycle studies (20 nM, A549-Luc, 24–72 h) showed a possible antiproliferative effect, although not statistically relevant, through arrest in the G1 phase, observed as an increase of the percentage of cells at this phase, and decrease of cells in the G2/M phase, relative to the AuNP@PEG and nontreated controls, that increases as the exposure time to the nanobeacons increases (Supporting Figure S5).

Taken together, Figure 4 establishes a mechanism-consistent selectivity triad - invasion suppressed, migration unchanged, viability preserved - that aligns with the platform’s miRNA targeting. An early functional sequestration of miR-31 by a minority cytosolic fraction of nanobeacons is sufficient to blunt ECM-dependent invasion by 72 h, while slower transcriptional feedback (documented later by the miR-31 time course) consolidates the effect without yielding a second step change at 120 h. This pattern provides a stringent benchmark for next-generation iterations, preserving migration/viability neutrality while shifting the anti-invasive response to earlier times and lower doses via enhanced endosomal escape.

### miR-31 Kinetics and Transcriptomic Remodeling

Time-resolved qRT-PCR in A549-Luc establishes a two-phase regulation of miR-31 after nanobeacon exposure. At 24–72 h, relative miR-31 levels fluctuate around control with high variance and no consistent depression (Figure 5A), indicating that the early anti-invasive phenotype (Figure 4) arises without a bulk decrease in mature miR-31. By 96 h the variance narrows, and by 120–144 h a clear reduction emerges, most evident at 20 nM and then apparent at both doses at 144 h, consistent with a transition from Phase I, in which nanobeacons functionally sequester a pool of miR-31 without measurably changing total abundance, to Phase II, in which transcriptional/processing feedback lowers mature miR-31. This timeline rationalizes the invasion data, where sequestration is already sufficient to blunt ECM-dependent invasion by 72 h, and once targets are largely derepressed, extending pretreatment to 120 h does not yield a second step-change in phenotype.

**Figure 5:**
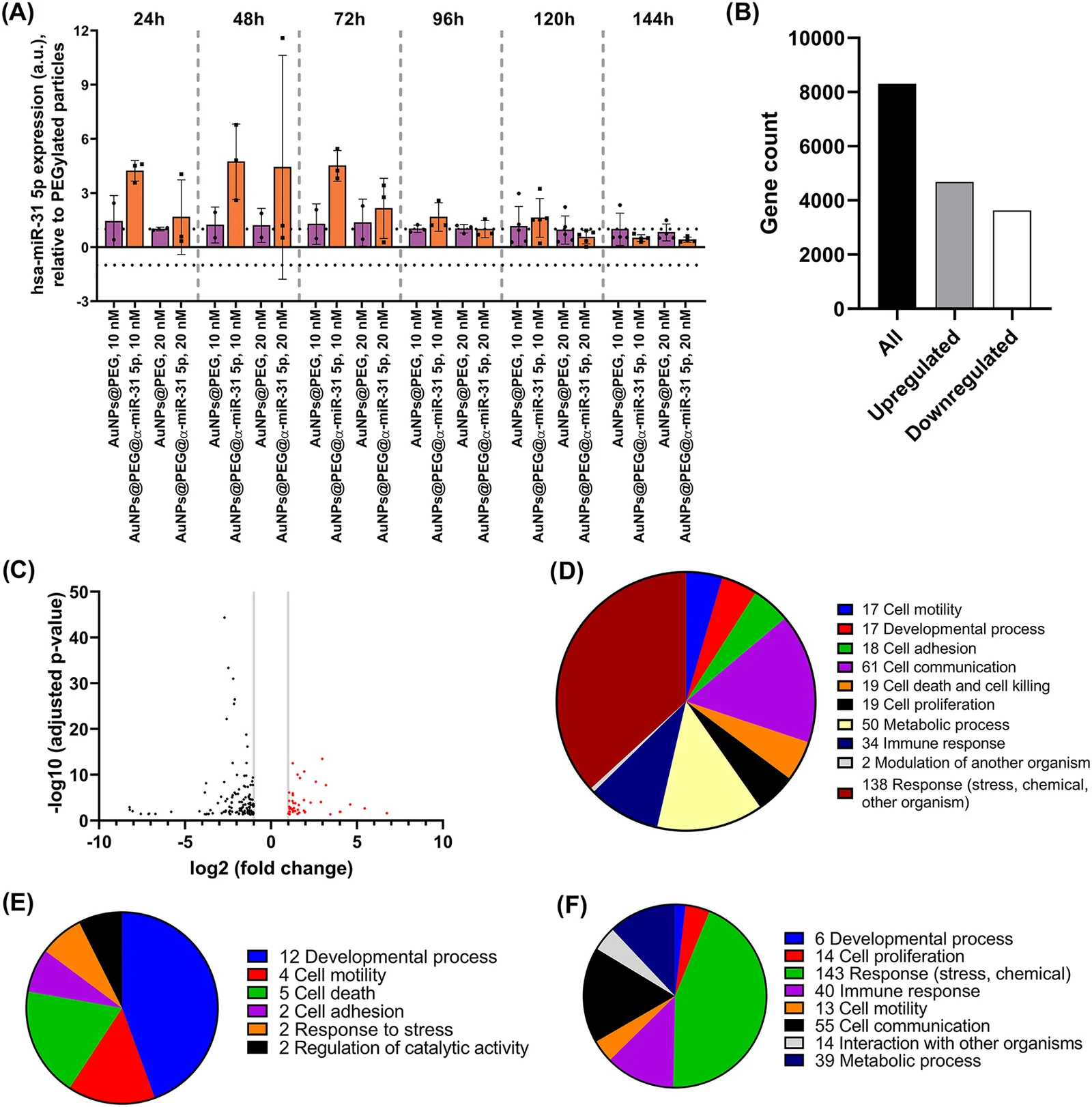
miR-31 kinetics and transcriptomic remodeling after nanobeacon treatment. (A) Time-course qRT-PCR of mature miR-31-5p in A549-Luc treated with AuNPs@PEG@α-miR-31-5p (10 or 20 nM) versus AuNPs@PEG controls at 24–144 h. Early time points (24–72 h) show high variance and no consistent decrease; a clear reduction emerges at 120–144 h, most evident at 20 nM. Bars represent the mean ± SD; symbols represent biological replicates. (B) RNA-seq summary at 72 h (20 nM): total detected genes and differentially expressed genes (DEGs; 46 up, 127 down). (C) Volcano plot of DEGs at 72 h showing modest-magnitude but statistically coherent shifts typical of miRNA-directed programs; red points denote significant DEGs (adjusted *p* < 0.05), gray band marks ± 1 log2 fold change. (D) Gene-ontology (GO) enrichment for all DEGs highlights processes linked to cell motility/adhesion, cell–cell/ECM communication, stress/immune response, cell death/proliferation, and metabolic processes. (E–F) GO partitions for up-regulated (E) and down-regulated (F) subsets emphasize motility/adhesion and developmental/EMT-related categories alongside stress/immune terms. Together, data supports a two-phase mechanism: early functional sequestration of miR-31 initiates network remodeling captured at 72 h by RNA-seq, followed by a measurable decline in mature miR-31 at 120–144 h that consolidates the selective anti-invasive phenotype.

Bulk transcriptomics at 72 h (20 nM) captures this early network remodeling before the late qRT-PCR drop. Of 17,906 expressed genes, 173 are differentially expressed (46 up, 127 down; Figure 5B). The volcano plot highlights modest-magnitude, statistically coherent changes (Figure 5C), typical of miRNA-directed programs. Gene-ontology analysis of all differentially expressed genes (DEGs) (Figure 5D) and of the up- and down-regulated subsets (Figure 5E,F) converges on categories central to the observed biology, that is, cell motility and adhesion, ECM and cell–cell communication, and stress/immune signaling, alongside cell-cycle terms. Notable candidates include adhesion and developmental/EMT nodes (e.g., *CDH4*, *WNT7A*), inflammatory/motility mediators (e.g., *CXCL8*), and cytoskeleton/contractility regulators (e.g., *S100A4*, *RGCC*), providing plausible axes through which miR-31 inhibition rewires the cell-ECM interface without depressing 2D migration. The enrichment pattern and directionality are consistent with derepression of miR-31 targets and secondary adjustments in their networks, yielding selective impairment of invasion while sparing planar motility and viability.

Mechanistically, the temporal offset between phenotype (decreased invasion at 72 h) and abundance (decreased miR-31 at 120–144 h) supports a sequestration-first model. Even a minority cytosolic fraction of delivered nanobeacons can titrate functionally available miR-31, initiating transcriptional programs that the 72 h RNA-seq already records. As feedback subsequently lowers miR-31 levels, the network shift consolidates rather than intensifies, mirroring the plateau in invasion between 72 and 120 h. Overall, our results tie molecular kinetics to systems-level remodeling, explaining how target-engaged nanobeacons yield a selective anti-invasive outcome that persists as miR-31 abundance later declines.

### Pathway-Level Transcriptomics Implicate Adhesion/ECM and Cytokine Programs

Unsupervised clustering of the 72 h RNA-seq profiles cleanly separates nanobeacon-treated A549-Luc cells from PEG-treated controls (Figure 6A), indicating a coordinated transcriptional response rather than isolated outliers; library composition is comparable between groups (Figure 6B). Differential analysis identifies a modest magnitude but statistically tight signature typical of miRNA perturbations (46 up, 127 down; Figure 6C). When the DEGs are organized by function, the pattern maps directly onto processes that miR-31 is known to influence, which are cell-matrix adhesion and traction generation, epithelial plasticity pathways, and inflammatory crosstalk.

**Figure 6:**
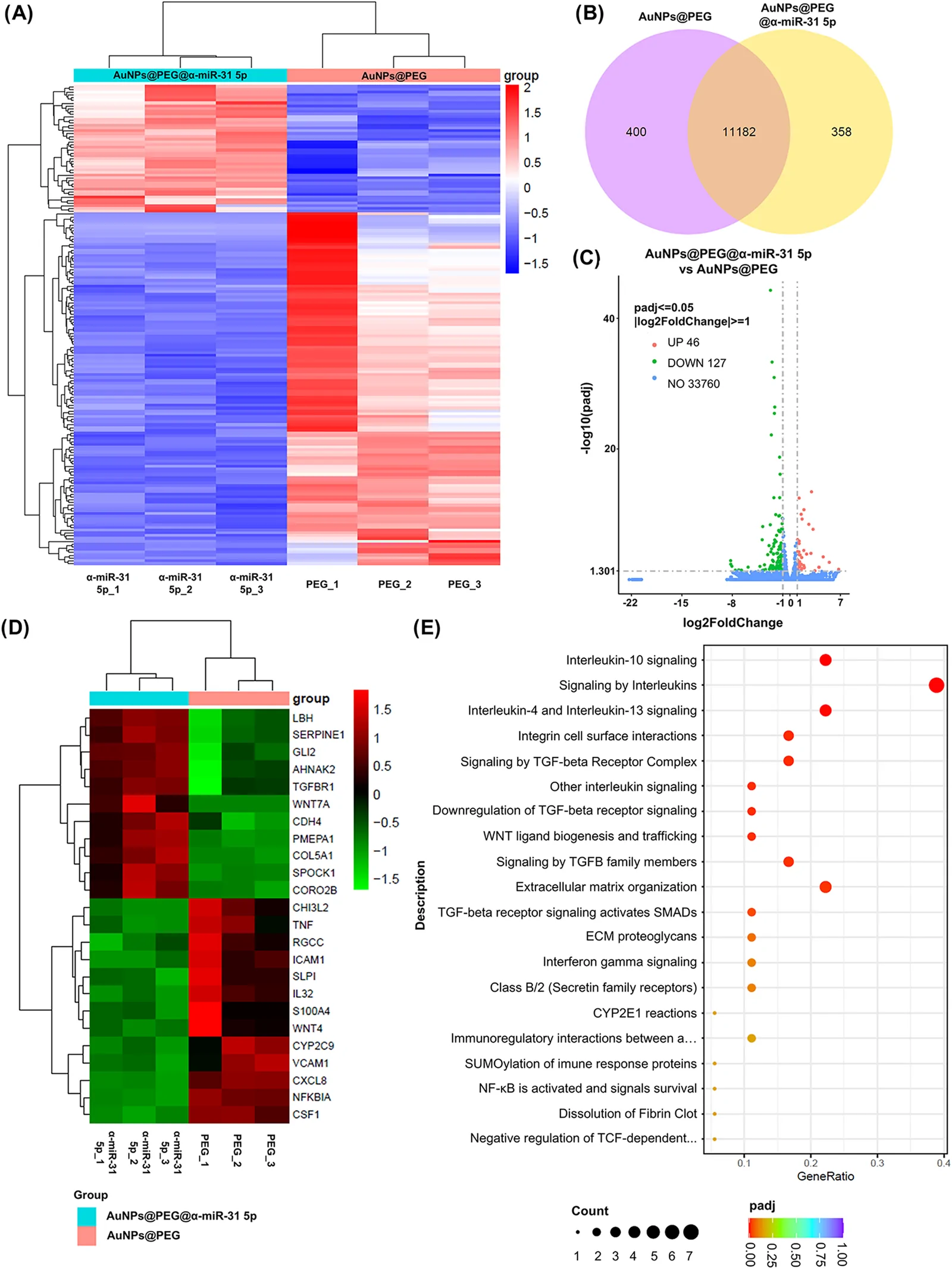
Pathway-level transcriptomics consistent with derepression of the miR-31 regulon (72 h). (A) Unsupervised clustering (variance-stabilized counts) separates AuNPs@PEG@α-miR-31-5p from AuNPs@PEG samples, indicating a coordinated response. (B) Library overlap of detected genes across groups; comparable composition supports downstream contrasts. (C) Differential expression (nanobeacon vs PEG) shows modest-magnitude but statistically tight miRNA-type signature (46 up, 127 down; padj <0.05; vertical lines mark |log2FC| = 1). (D) Curated heat map of invasion-relevant genes highlights concerted shifts in adhesion/ECM and cytoskeletal regulators (e.g., *CDH4*, *COL5A1*, *SPOCK1*, *CORO2B*), developmental/EMT nodes (*WNT7A*/*WNT4*, *GLI2*, *TGFBR1*, *PMEPA1*, *LBH*), motility/contractility markers (*S100A4*, *RGCC*), and inflammatory/vascular interactors (*ICAM1*, *VCAM1*, *CXCL8*, *TNF*, *CSF1*, *NFKBIA*). Several are reported or predicted miR-31 targets/proximal nodes, and the directionality (seed-bearing transcripts up) is consistent with miR-31 inhibition. (E) GO/Reactome enrichment emphasizes integrin cell-surface interactions, extracellular matrix organization, TGF-β/WNT signaling, and interleukin/NF-κB/IFN-γ modules, pathways that govern ECM-dependent invasion rather than planar migration. Together, the 72 h transcriptome reveals early rewiring of miR-31-controlled adhesion/EMT/cytokine circuits prior to the bulk decrease in mature miR-31 (Figure 5), supporting a sequestration-first mechanism that underlies the selective anti-invasive phenotype.

At the gene level (Figure 6D), adhesion/ECM and cytoskeletal regulators (*CDH4*, *COL5A1*, *SPOCK1*, *CORO2B*) move in concert with developmental/EMT nodes (*WNT7A*/*WNT4*, *GLI2*, *TGFBR1*, *PMEPA1*, *LBH*) and motility/contractility markers (*S100A4*, *RGCC*), alongside endothelial/immune interactors (*ICAM1*, *VCAM1*, *CXCL8*, *TNF*, *CSF1*, *NFKBIA*). Several of these genes have been reported as direct or context-proximal miR-31 targets/neighbors in the literature, and, critically, the direction of change is consistent with miR-31 inhibition. Transcripts predicted to carry conserved miR-31 seed sites tend to be upregulated, while counter-regulatory nodes downstream of these targets exhibit compensatory decreases. In other words, the transcriptome looks like derepression of the miR-31 regulon overlaid with secondary network adjustment.

Pathway enrichment consolidates this view (Figure 6E). Terms with the strongest support include integrin cell-surface interactions and extracellular matrix organization (adhesion/traction), TGF-β receptor and WNT signaling (epithelial plasticity/EMT control), and interleukin/NF-κB/IFN-γ modules (paracrine remodeling). This cluster is exactly what would follow functional sequestration of miR-31, that is, immediate relief of post-transcriptional repression on adhesion/ECM and signaling hubs, with downstream tuning of cytokine circuits that shape the invasive program. The timing is also diagnostic. We observe these pathway shifts already at 72 h, before the bulk drop in mature miR-31 (Figure 5A), which supports a sequestration-first mechanism in which only a minor cytosolic fraction of nanobeacons needs to bind miR-31 to rewire phenotype-defining modules. As feedback later reduces miR-31 abundance (120–144 h), the network state consolidates rather than produces a second jump, matching the invasion plateau between 72 and 120 h.

Altogether, we showed that target-engaged inhibition of miR-31 does not induce a generic stress response; it reprograms the miR-31 regulon and its immediate circuits, adhesion/ECM mechanics, EMT-linked signaling, and cytokine crosstalk, providing a mechanistic bridge from nanobeacon chemistry to the selective anti-invasion phenotype with preserved migration and viability.

### *In Vivo* Histology Reveals Local Microenvironment Remodeling

End point histology provides spatial confirmation that the nanobeacons persist within tumors and coincide with regional microenvironmental changes. H&E sections from control (HEC@AuNPs@PEG) and treated (HEC@AuNPs@PEG@α-miR-31-5p) tumors show well-defined tumor-stroma borders, with dense epithelial nests abutting eosinophilic stromal bands (Figure 7A–B). Within these interfaces, and particularly at tumor rims, black, granular deposits are frequently observed (boxed regions in A–B; magnified in C–D). Their morphology (irregular, highly refractile granules) is characteristic of gold aggregates, and they are embedded among small, round inflammatory cells consistent with myeloid infiltrates, suggesting local retention and phagocytic uptake of the nanoconjugates. These features occur in both groups, as expected for locally injected gold formulations, but they are more conspicuous in treated specimens, consistent with the beacon-bearing surface chemistry favoring tissue retention.

**Figure 7:**
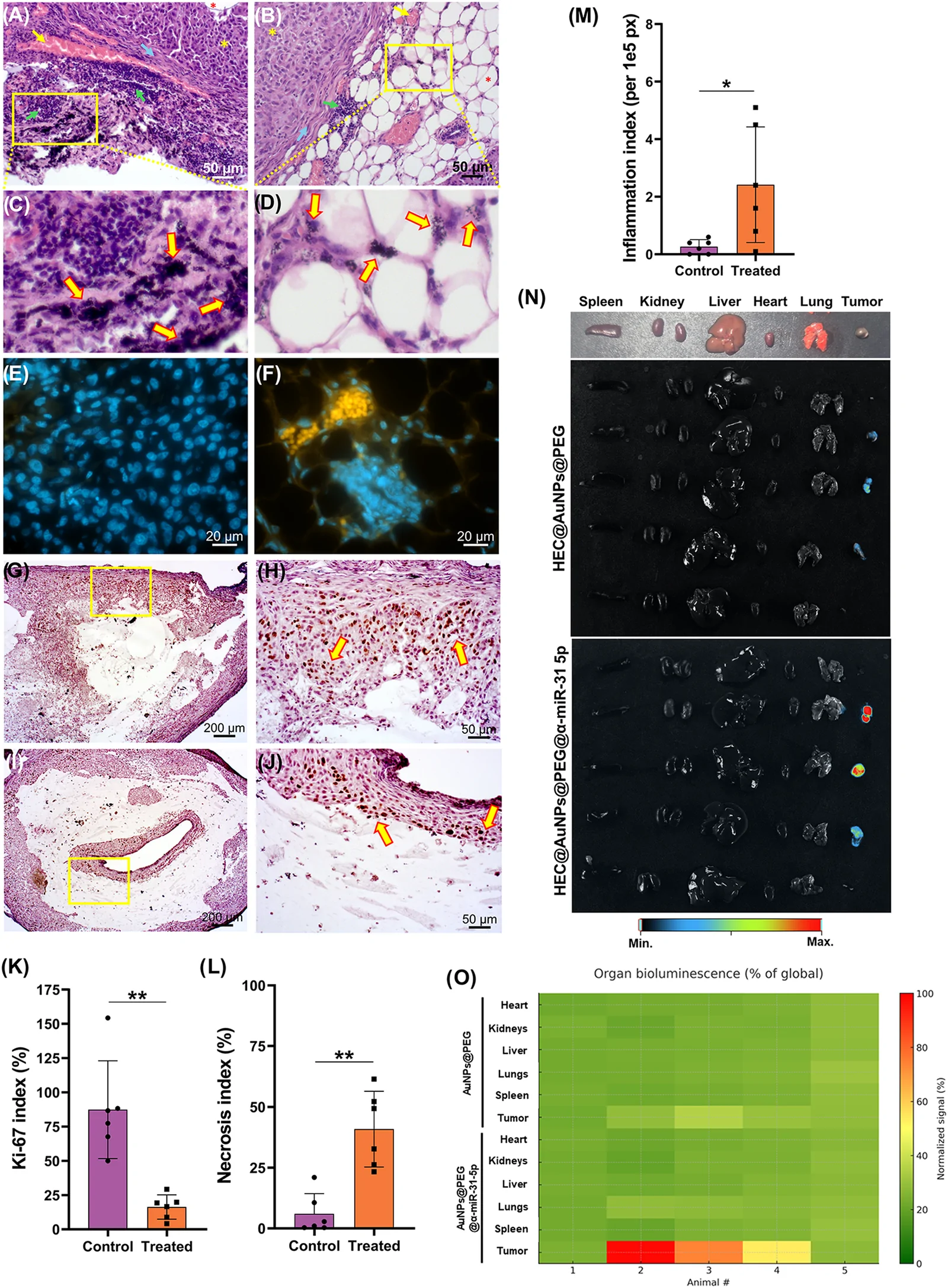
*In vivo* histology reveals local microenvironment remodeling. (A–B) H&E sections showing tumor-stroma interfaces in control HEC@AuNPs@PEG (A) and treated HEC@AuNPs@PEG@α-miR-31-5p (B) tumors. Boxed regions mark areas enlarged in (C–D). Black, granular deposits at the interface and within stroma are morphologically consistent with gold aggregates and are surrounded by inflammatory cells. Scale bars: 50 μm. (C–D) Higher magnification of boxed regions from (A–B) highlighting the black deposits (arrows) within inflamed stroma/adipose septa, suggesting local retention and phagocytic uptake. (E–F) Immunofluorescence: control tumor shows DAPI-stained nuclei only (E); treated tumor exhibits Cy3-bright clusters (F) consistent with beacon-bearing AuNPs retained in stromal pockets. Scale bars: 20 μm. (G–J) *K*_i_-67 immunohistochemistry. Control tumors display a peripheral proliferative rim with lower central labeling (G, H). Treated tumors show a heterogeneous pattern with focal reductions in *K*_i_-67 within stromal/fibrotic regions (I, J), indicating microdomain-level remodeling near particle deposits. Scale bars: 200 μm (G, I) and 50 μm (H, J). (K) Quantification of the *K*_i_-67 index, evidencing lower proliferation on HEC@AuNPs@PEG@α-miR-31–5p-treated tumors. (L) Quantification of necrosis, showing higher levels upon nanobeacon treatment. (M) Inflammation index, correlating the presence of immune infiltrates with higher inflammation in nanobeacon-treated tumors. Bars show mean ± SD; symbols denote independent replicates; significance markers on bar charts correspond to the Welch’s *t* test (*p < 0.05; **p < 0.01). (N) *Ex vivo* bioluminescence of organs and tumors at end point under identical IVIS settings (top: control; bottom: treated). Signals are restricted to tumors; spleen, kidney, liver, heart, and lung are in the background. Color bar: radiance from low (blue) to high (red). (O) *Ex vivo* bioluminescence signal quantification. Color bar: normalized signal from 0% (green) to 100% (red). Together, the panels show intratumoral persistence of beacon-bearing AuNPs, association with innate immune-like infiltrates, localized decreases in proliferation and increased necrosis and inflammation, consistent with microenvironment remodeling.

Direct immunofluorescence corroborates nanoparticle presence. Control tumors show only nuclear DAPI (Figure 7E), whereas treated tumors display Cy3-bright clusters (Figure 7F) that colocalize with stromal pockets and cell aggregates. The spatial match between Cy3 clusters (Figure 7F) and H&E black deposits (Figure 7A–D) supports the interpretation that the fluorescent signal originates from beacon-bearing AuNPs rather than tissue autofluorescence, and that these particles persist within stromal/vascular niches at end point.

Proliferative architecture, assessed by *K*_i_-67 IHC, differs between groups in a manner consistent with localized remodeling. Controls exhibit the classic peripheral proliferative rim with low labeling toward the core (Figure 7G–H), often adjacent to gold deposits. Treated tumors show a heterogeneous mosaic, with islands of *K*_i_-67-positive cells encircling central zones of reduced *K*_i_-67 and fibrotic stroma rather than frank necrosis (Figure 7I–J). The reduction is focal, not a global suppression, implying microdomain-level effects in regions of high particle exposure. Mechanistically, this pattern is compatible with beacon-driven modulation of stromal and cytokine circuits (as suggested by the 72 h RNA-seq) and with matrix remodeling that disfavors proliferation locally. Quantification of the *K*_i_-67 index shows that HEC@AuNPs@PEG@α-miR-31-5p-treated tumors have less proliferative areas than HEC@AuNPs@PEG-treated tumors (*K*_i_-67 index 0–50% vs 50–100%) (Figure 7K). Additionally, HEC@AuNPs@PEG@α-miR-31-5p-treated tumors showed a higher necrosis percentage (Figure 7L) and inflammatory index (Figure 7M), evidencing immune-cell recruitment to the tumor site in response to Au nanobeacons.

Organ-wide safety and tumor localization of the model readout are supported by *ex vivo* bioluminescence (Figure 7N–O). Under identical settings, radiance is restricted to tumors in both groups, with spleen, kidneys, liver, heart, and lungs in the background. While bioluminescence does not report nanoparticle distribution, the tumor-only signal complements histology by excluding detectable ectopic luciferase activity and reinforcing localized disease burden at end point. Moreover, the nanobeacons proved to be safe and biocompatible in both groups, as the mice weight did not show unexpected variations throughout the experiment, and there were no signs that would imply early euthanasia (Supporting Figure S6).

Taken together, our results show that beacon-bearing AuNPs persist intratumorally, associate with innate-immune-like infiltrates and coincide with regional decreases in proliferation, fibrotic remodeling, necrosis and inflammation, a spatial signature of local microenvironment reprogramming that is coherent with the selective anti-invasive biology observed *in vitro*. These observations motivate quantitative follow-ups, with multiplex IF/IHC for macrophage/neutrophil/endothelial markers, and collagen mapping, to formally link nanobeacon loci with stromal and proliferative states.

## Conclusions

In conclusion, PEG–gold nanobeacons targeting miR-31-5p establish a direct link between rational materials design and selective phenotypic control. Despite predominant lysosomal trafficking, these constructs achieve early functional sequestration of miR-31 that is sufficient to suppress ECM-dependent invasion without affecting viability or planar migration, with efficacy scaling to basal miR-31 levels. This early effect precedes a delayed transcriptional feedback phase marked by reduced miR-31 abundance and coordinated remodeling of adhesion, ECM, and signaling programs. *In vivo*, nanobeacons persist locally within tumors and associate with focal reductions in proliferation and increased necrotic and inflammatory features, without off-target burden. Together, these results demonstrate that readout-enabled anti-miRNA nanobeacons provide a modular, noncytotoxic strategy to reprogram invasive behavior and define a physicochemical and mechanistic framework for further optimization and translation.

## Supplementary Material



## Data Availability

The data sets used and/or analyzed during the current study are available from the corresponding author on reasonable request.

## Notes

Additional results and experimental details: tables containing data on the oligonucleotides used, miRNA array results and differentially expressed genes; data sets relative to the optimization of the functionalization process, additional characterization assays of the nanobeacon, TEM photographs of the control nanoparticles, additional representations of the migration and invasion results mentioned in the manuscript, data relative to a cell cycle assay, and information regarding the mouse weight and the IHC controls used. It also includes the gating strategy used in the flow cytometry assays performed (PDF)

## References

[1] de
Visser K. E., Joyce J. A. (2023). The Evolving Tumor Microenvironment:
From Cancer Initiation to Metastatic Outgrowth. Cancer Cell.

[2] Baghban R., Roshangar L., Jahanban-Esfahlan R., Seidi K., Ebrahimi-Kalan A., Jaymand M., Kolahian S., Javaheri T., Zare P. (2020). Tumor Microenvironment
Complexity and Therapeutic Implications at a Glance. Cell Commun. Signaling.

[3] O’Brien J., Hayder H., Zayed Y., Peng C. (2018). Overview of MicroRNA
Biogenesis, Mechanisms of Actions, and Circulation. Front. Endocrinol..

[4] Chatterjee M., Nag S., Gupta S., Mukherjee T., Shankar P., Parashar D., Maitra A., Das K. (2025). MicroRNAs
in Lung Cancer: Their Role
in Tumor Progression, Biomarkers, Diagnostic, Prognostic, and Therapeutic
Relevance. Discover Oncol..

[5] Yu T., Ma P., Wu D., Shu Y., Gao W. (2018). Functions and Mechanisms
of MicroRNA-31 in Human Cancers. Biomed. Pharmacother..

[6] Cottonham C. L., Kaneko S., Xu L. (2010). MiR-21 and
MiR-31 Converge on TIAM1
to Regulate Migration and Invasion of Colon Carcinoma Cells. J. Biol. Chem..

[7] Liu M., Bi F., Zhou X., Zheng Y. (2012). Rho GTPase Regulation by MiRNAs and
Covalent Modifications. Trends Cell Biol..

[8] Stepicheva N. A., Song J. L. (2016). Function and Regulation of MicroRNA-31 in Development
and Disease. Mol. Reprod. Dev..

[9] Meng W., Ye Z., Cui R., Perry J., Dedousi-Huebner V., Huebner A., Wang Y., Li B., Volinia S., Nakanishi H., Kim T., Suh S.-S., Ayers L. W., Ross P., Croce C. M., Chakravarti A., Jin V. X., Lautenschlaeger T. (2013). MicroRNA-31 Predicts the Presence
of Lymph Node Metastases and Survival in Patients with Lung Adenocarcinoma. Clin. Cancer Res..

[10] Wang Y., Shang S., Yu K., Sun H., Ma W., Zhao W. (2020). MiR-224, MiR-147b and MiR-31 Associated
with Lymph Node Metastasis
and Prognosis for Lung Adenocarcinoma by Regulating PRPF4B, WDR82
or NR3C2. PeerJ.

[11] Zhu C., Wang S., Zheng M., Chen Z., Wang G., Ma J., Zhang B., Huang W., Sun X., Wang C. (2022). MiR-31–5p
Modulates Cell Progression in Lung Adenocarcinoma through TNS1/P53
Axis. Strahlenther. Onkol..

[12] Hao Z., Zhang M., Du Y., Liu J., Zeng G., Li H., Peng X. (2025). Invadopodia in Cancer
Metastasis: Dynamics, Regulation,
and Targeted Therapies. J. Transl. Med..

[13] Esau C. C. (2008). Inhibition
of MicroRNA with Antisense Oligonucleotides. Methods.

[14] Lima J. F., Cerqueira L., Figueiredo C., Oliveira C., Azevedo N. F. (2018). Anti-MiRNA
Oligonucleotides: A Comprehensive Guide for Design. RNA Biol..

[15] Liu M., Wang Y., Zhang Y., Hu D., Tang L., Zhou B., Yang L. (2025). Landscape of Small Nucleic Acid Therapeutics:
Moving from the Bench to the Clinic as next-Generation Medicines. Signal Transduction Targeted Ther..

[16] Duman H., Akdaşçi E., Eker F., Bechelany M., Karav S. (2024). Gold Nanoparticles:
Multifunctional Properties, Synthesis, and Future
Prospects. Nanomaterials.

[17] Arcos
Rosero W. A., Bueno Barbezan A., Daruich de Souza C., Chuery Martins Rostelato M. E. (2024). Review of Advances in Coating and
Functionalization of Gold Nanoparticles: From Theory to Biomedical
Application. Pharmaceutics.

[18] Tiwari P. M., Vig K., Dennis V. A., Singh S. R. (2011). Functionalized Gold Nanoparticles
and Their Biomedical Applications. Nanomaterials.

[19] Dutour R., Bruylants G. (2025). Gold Nanoparticles Coated with Nucleic Acids: An Overview
of the Different Bioconjugation Pathways. Bioconjugate
Chem..

[20] Monroy-Contreras R., Vaca L. (2011). Molecular Beacons:
Powerful Tools for Imaging RNA in Living Cells. J. Nucleic Acids.

[21] Sousa D. P., Conde J. (2022). Using Gold Nanobeacons as a Theranostic Technique to Recognize, Detect,
and Inhibit Specific Nucleic Acids. STAR Protoc..

[22] Wang X., Li H., Chen C., Liang Z. (2025). Understanding of Endo/Lysosomal Escape
of Nanomaterials in Biomedical Application. Smart Mol..

[23] Grau M., Wagner E. (2024). Strategies and Mechanisms for Endosomal Escape of Therapeutic
Nucleic Acids. Curr. Opin. Chem. Biol..

[24] Kilchrist K. V., Dimobi S. C., Jackson M. A., Evans B. C., Werfel T. A., Dailing E. A., Bedingfield S. K., Kelly I. B., Duvall C. L. (2019). Gal8 Visualization
of Endosome Disruption Predicts Carrier-Mediated Biologic Drug Intracellular
Bioavailability. ACS Nano.

[25] Nayanathara U., Yang F., Zhang C., Wang Y., Rossi Herling B., Smith S. A., Beach M. A., Johnston A. P. R., Such G. K. (2025). Unravelling
the Endosomal Escape of PH-Responsive Nanoparticles Using the Split
Luciferase Endosomal Escape Quantification Assay. Biomater. Sci..

[26] Oliveros, J. C. VENNY. An interactive tool for comparing lists with Venn Diagrams, version 2.1.0, http://bioinfogp.cnb.csic.es/tools/venny/index.html.

[27] Agarwal V., Bell G. W., Nam J.-W., Bartel D. P. (2015). Predicting
Effective
MicroRNA Target Sites in Mammalian MRNAs. Elife.

[28] Dennis G., Sherman B. T., Hosack D. A., Yang J., Gao W., Lane H. C., Lempicki R. A. (2003). DAVID:
Database for Annotation, Visualization,
and Integrated Discovery. Genome Biol..

[29] Sherman B. T., Hao M., Qiu J., Jiao X., Baseler M. W., Lane H. C., Imamichi T., Chang W. (2022). DAVID: A Web
Server for Functional
Enrichment Analysis and Functional Annotation of Gene Lists (2021
Update). Nucleic Acids Res..

[30] Edmonds M. D., Boyd K. L., Moyo T., Mitra R., Duszynski R., Arrate M. P., Chen X., Zhao Z., Blackwell T. S., Andl T., Eischen C. M. (2016). MicroRNA-31
Initiates Lung Tumorigenesis
and Promotes Mutant KRAS-Driven Lung Cancer. J. Clin. Invest..

[31] Yu F., Liang M., Huang Y., Wu W., Zheng B., Chen C. (2021). Hypoxic Tumor-Derived Exosomal MiR-31–5p Promotes Lung Adenocarcinoma
Metastasis by Negatively Regulating SATB2-Reversed EMT and Activating
MEK/ERK Signaling. J. Exp. Clin. Cancer Res..

[32] Ren J. (2023). Intermittent
Hypoxia BMSCs-Derived Exosomal MiR-31–5p Promotes Lung Adenocarcinoma
Development via WDR5-Induced Epithelial Mesenchymal Transition. Sleep Breathing.

[33] Zhang X.-D., Wu D., Shen X., Liu P.-X., Fan F.-Y., Fan S.-J. (2012). In Vivo
Renal Clearance, Biodistribution, Toxicity of Gold Nanoclusters. Biomaterials.

[34] Xu M., Qi Y., Liu G., Song Y., Jiang X., Du B. (2023). Size-Dependent
In Vivo Transport of Nanoparticles: Implications for Delivery, Targeting,
and Clearance. ACS Nano.

