# Supplementary material for: DNA/RNA Hybrid Hairpin Gold Nanobeacons Targeting miR-31 Reprogram Invasion in Lung Cancer and Remodel Tumor Histo-Architecture In Vivo
Source: ACS Mater Au. 2026 Apr 7;6(5):1046–63. doi: 10.1021/acsmaterialsau.5c00231 (PMC13564150; doi:10.1021/acsmaterialsau.5c00231)
Supplement: Supplementary file 1 [file mg5c00231_si_001.pdf]

Supporting Information

DNA/RNA hybrid hairpin gold nanobeacons targeting miR-31 reprogram invasion in lung cancer and remodel tumor histo-architecture *in vivo*

Diana P. Sousa[1,2], Jhenifer Oliveira[1,2,#], Catarina F. Martins[1,2,#] and João Conde[1,2,*]

[1] NOVA Medical School|Faculdade de Ciências Médicas, NMS|FCM, Universidade NOVA de Lisboa, 1169-056 Lisbon, Portugal.
[2] Comprehensive Health Research Centre (CHRC), NOVA Medical School|Faculdade de Ciências Médicas, NMS|FCM, Universidade NOVA de Lisboa, 1169-056 Lisbon, Portugal.

# These authors contributed equally to this paper.
* Corresponding author: joao.conde@nms.unl.pt

Supplementary Tables and Figures

Table S1 - Oligonucleotides used for nanoparticle functionalization and for target hybridization assays.

Oligonucleotide	Sequence*	Length (# of nucleotides)	Purification	Molecular Weight (g/mol)	T_m (ºC)	GC (%)	Modifications	Notes
**Anti-miR-31 5p**	5' **TTT GCC** - AGC UAU GCC AGC AUC UUG CCU - **GGC AAA** 3'	33	HPLC	11324.4	53.1	33.3	5' Thiol, 3' Cyanine3	Sequence used to functionalize AuNPs
**Anti-miR-31 3p**	5' **TTT GCC** - UAC CGU UAU ACA ACC GUA UCG U - **GGC AAA** 3'	34	HPLC	11336	59.1	44.1	5' Thiol, 3' Cyanine3	Sequence used to functionalize AuNPs
**hsa-miR-31 5p**	5' AGG CAA GAU GCU GGC AUA GCU 3'	21	HPLC	6776	66	52.3	None	miRNA target sequence, target hybridization assays
**hsa-miR-31 3p**	5' UGC UAU GCC AAC AUA UUG CCA U – 3'	22	HPLC	6925	60.7	40.9	None	
**Irrelevant control**	5' CCG UUA CUA UUU GCA CCA C 3'	19	HPLC	5919	45	47	None	Irrelevant control sequence, target hybridization assays

*Nucleotides in **bold** correspond to DNA; Nucleotides without bold correspond to RNA

Table S2 – Top 100 differentially expressed miRNAs obtained in the miRNA arrays of A549-Luc and RERF LC Sq1 CMV-Luc cells and used for target selection.

miRNA name	A549-Luc log2 (Fold Change)	RERF LC Sq1 CMV-Luc log2 (Fold Change)	Adjusted p-value (padj)
hsa-miR-205-5p	0.25	13.63	$2.11x10^{-14}$
hsa-miR-31-5p	11.88	0.39	$4.51x10^{-13}$
hsa-miR-200b-5p	0.57	7.14	$2.73x10^{-10}$
hsa-miR-200b-3p	0.44	8.01	$3.64x10^{-10}$
hsa-miR-200c-3p	5.07	13.12	$5.45x10^{-10}$
hsa-miR-504-5p	0.36	6.81	$6.91x10^{-9}$
hsa-miR-760	3.2	7.6	$1.31x10^{-8}$
hsa-miR-1269b	5.5	0.4	$1.72x10^{-8}$
hsa-miR-6510-3p	0.33	4.7	$2.51x10^{-8}$
hsa-miR-708-5p	1.9	6.98	$3.70x10^{-8}$
hsa-miR-203a	1.08	4.83	$6.60x10^{-8}$
hsa-miR-92b-3p	9.95	6.95	$7.07x10^{-8}$
hsa-miR-200a-3p	0.2	5.52	$8.71x10^{-8}$
hsa-miR-3620-3p	1.14	4.88	$9.49x10^{-8}$
hsa-miR-363-3p	0.31	4.01	$9.70x10^{-8}$
hsa-miR-625-5p	4.94	8.25	$1.12x10^{-7}$
hsa-miR-145-5p	1.02	5.22	$1.12x10^{-7}$
hsa-miR-615-3p	5.15	7.99	$1.38x10^{-7}$
hsa-miR-424-3p	8.48	5.63	$1.47x10^{-7}$
hsa-miR-432-5p	3.76	0.5	$1.70x10^{-7}$
hsa-miR-187-3p	0.3	3.78	$1.70x10^{-7}$
hsa-miR-193b-5p	8.19	4.64	$1.85x10^{-7}$
hsa-miR-4713-5p	0.83	4.69	$2.39x10^{-7}$
hsa-miR-196b-5p	1.72	5.75	$2.47x10^{-7}$
hsa-miR-127-3p	4.66	0.68	$2.88x10^{-7}$
hsa-miR-34c-3p	1.67	5.01	$2.88x10^{-7}$
hsa-mir-92b	7.28	4.71	$3.22x10^{-7}$
hsa-miR-3200-3p	2.33	5.98	$4.07x10^{-7}$
hsa-miR-874-3p	3.53	8.04	$4.97x10^{-7}$
hsa-miR-1299	4.18	0.84	$5.41x10^{-7}$
hsa-miR-505-5p	4.73	7.84	$8.52x10^{-7}$
hsa-miR-378g	2.2	5.05	$1.48x10^{-6}$
hsa-miR-935	8.18	5.83	$1.53x10^{-6}$
hsa-miR-152-3p	1.39	6.99	$1.62x10^{-6}$
hsa-miR-193b-3p	12.83	10.71	$1.76x10^{-6}$
hsa-miR-181a-5p	10.91	8.86	$1.76x10^{-6}$
hsa-miR-197-3p	8.21	10.35	$2.38x10^{-6}$
hsa-miR-1247-5p	3.43	0.74	$3.25x10^{-6}$
hsa-miR-452-5p	1.06	6.64	$3.46x10^{-6}$

hsa-miR-326	1.03	3.78	$3.52x10^{-6}$
hsa-miR-1226-3p	3.11	6.34	$3.91x10^{-6}$
hsa-miR-3065-3p	0.34	2.68	$4.28x10^{-6}$
hsa-miR-194-3p	5.24	1.8	$4.28x10^{-6}$
hsa-miR-378a-3p	7.65	9.91	$5.23x10^{-6}$
hsa-miR-503-5p	6.7	4.13	$5.92x10^{-6}$
hsa-miR-491-5p	3.92	0.39	$7.91x10^{-6}$
hsa-miR-224-3p	6.18	8.13	$8.92x10^{-6}$
hsa-miR-204-3p	1.89	4.74	$9.54x10^{-6}$
hsa-miR-192-5p	5.08	0.84	$9.54x10^{-6}$
hsa-miR-149-5p	7.73	9.64	$1.17x10^{-5}$
hsa-miR-29b-1-5p	7.27	5.47	$1.24x10^{-5}$
hsa-miR-3065-5p	0.34	3.53	$1.55x10^{-5}$
hsa-miR-1269a	3.73	0.58	$1.75x10^{-5}$
hsa-miR-487b-3p	2.96	0.04	$1.95x10^{-5}$
hsa-miR-574-3p	7.63	9.25	$2.48x10^{-5}$
hsa-miR-200a-5p	0.65	3.79	$2.71x10^{-5}$
hsa-miR-874-5p	1.05	3.75	$3.37x10^{-5}$
hsa-miR-378c	6.01	7.98	$3.43x10^{-5}$
hsa-miR-378d	3.27	5.73	$3.97x10^{-5}$
hsa-miR-6499-5p	0.54	2.8	$4.16x10^{-5}$
hsa-miR-1910-5p	4.66	6.65	$4.56x10^{-5}$
hsa-miR-139-5p	2.45	5.28	$4.80x10^{-5}$
hsa-miR-422a	5.47	7.25	$4.84x10^{-5}$
hsa-miR-194-5p	7.95	4.18	$5.03x10^{-5}$
hsa-miR-877-5p	4.97	7.76	$5.03x10^{-5}$
hsa-miR-141-3p	0.65	2.84	$5.03x10^{-5}$
hsa-miR-181b-5p	9.97	7.75	$5.03x10^{-5}$
hsa-miR-6779-5p	8.35	6.11	$5.03x10^{-5}$
hsa-miR-138-5p	7.25	5.18	$5.03x10^{-5}$
hsa-miR-125b-2-3p	3.16	0.71	$5.53x10^{-5}$
hsa-mir-1254-2	4.09	2.55	$5.76x10^{-5}$
hsa-miR-1254	5.37	3.25	$6.12x10^{-5}$
hsa-miR-628-3p	2.21	4.76	$6.13x10^{-5}$
hsa-miR-1910-3p	2.03	4.49	$6.32x10^{-5}$
hsa-miR-92a-1-5p	5.75	2.14	$6.42x10^{-5}$
hsa-miR-1229-3p	1.46	4.92	$6.81x10^{-5}$
hsa-miR-455-3p	8.39	6.85	$6.81x10^{-5}$
hsa-miR-378i	4.71	6.71	$7.04x10^{-5}$
hsa-miR-486-5p	5.68	4.01	$7.04x10^{-5}$
hsa-miR-934	0.25	2.75	$9.21x10^{-5}$
hsa-miR-4787-3p	1.37	3.85	$1.00x10^{-4}$
hsa-miR-505-3p	3.6	6.69	$1.00x10^{-4}$
hsa-miR-769-5p	3.3	5.71	$1.00x10^{-4}$

hsa-miR-584-5p	1.93	4.67	$1.00x10^{-4}$
hsa-mir-31	3.57	0.88	$2.00x10^{-4}$
hsa-miR-6872-5p	1.12	3.41	$2.00x10^{-4}$
hsa-miR-182-5p	7.65	9.36	$2.00x10^{-4}$
hsa-miR-106b-5p	7.53	9.23	$3.00x10^{-4}$
hsa-miR-193a-5p	8.68	7.03	$3.00x10^{-4}$
hsa-miR-330-3p	5.4	6.94	$3.00x10^{-4}$
hsa-miR-1287-5p	0.91	3.35	$3.00x10^{-4}$
hsa-miR-99b-3p	6.97	8.39	$4.00x10^{-4}$
hsa-miR-6784-5p	5.17	2.62	$4.00x10^{-4}$
hsa-miR-23a-5p	7.22	5.46	$4.00x10^{-4}$
hsa-miR-93-3p	4.93	6.7	$4.00x10^{-4}$
hsa-miR-183-3p	4.08	5.97	$4.00x10^{-4}$
hsa-miR-1306-5p	2.27	4.32	$5.00x10^{-4}$
hsa-miR-296-3p	5.23	6.78	$5.00x10^{-4}$
hsa-miR-204-5p	0.56	2.29	$5.00x10^{-4}$
hsa-miR-183-5p	6.26	8.18	$5.00x10^{-4}$

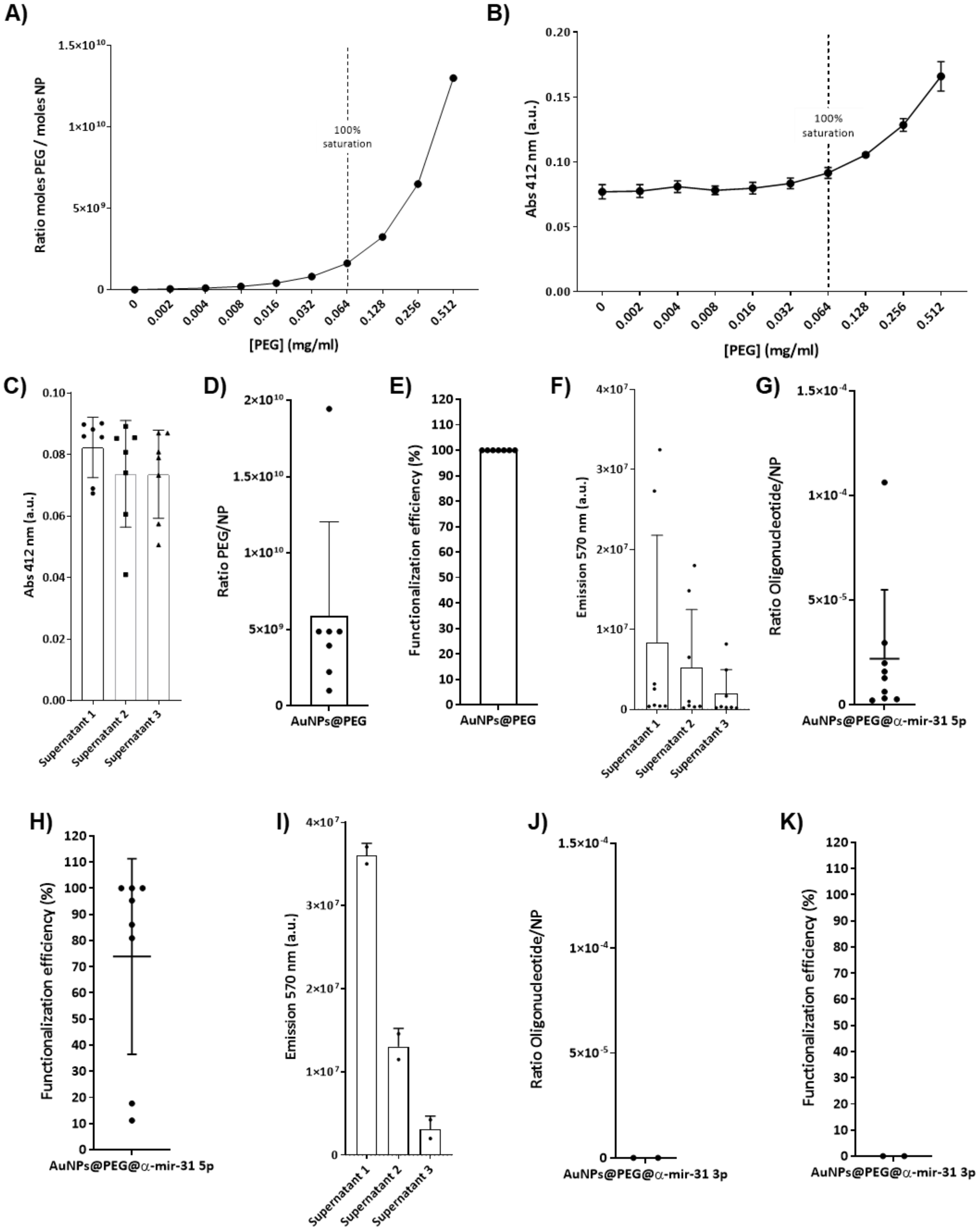

Figure S1 – Construction optimization and scale-up of gold nanobeacons. (A) Optimization of the PEG concentration on 60 nm AuNPs: absorbance at 412 nm obtained from the colorimetric assay using Ellman's reaction, indicative of the presence of free thiols in solution, in function of increasing PEG concentrations added to the nanoparticles. (B) Ratio moles of PEG:moles of AuNPs obtained by estimating the moles of PEG in the nanoparticles after calculation of the moles of free thiols in the solution at the different PEG concentrations. (C) Scale up of the functionalization of 60 nm AuNPs@PEG:

absorbance at 412 nm obtained from the colorimetric assay using Ellman's reaction after analysis of the supernatants collected throughout the scaling up process of AuNP PEGylation. (D) Ratio moles of PEG:moles of AuNPs obtained by estimating the moles of free thiolated PEG in solution after functionalization, by measuring the absorbance at 412 nm after using the Ellman's reaction. (E) Functionalization efficiency of the AuNPs with PEG upon scaling up the process. (F) Supernatant analysis by evaluation of the fluorescence emission (570 nm) of the AuNPs@PEG@α-miR-31-5p. (G) Ratio moles of oligonucleotide:moles of AuNPs obtained by estimating the moles of free molecular beacon (α-miR-31-5p) in solution after functionalization, by measuring fluorescence of the supernatant. (H) Functionalization efficiency of the AuNPs@PEG with α-miR-31-5p. (I) Supernatant analysis by fluorescence emission (570 nm) of the AuNPs@PEG@α-miR-31-3p. (J) Ratio moles of oligonucleotide:moles of AuNPs obtained by estimating the moles of free molecular beacon (α-miR-31-3p) in solution after functionalization, by measuring fluorescence of the supernatant. (K) Functionalization efficiency of the AuNPs@PEG with α-miR-31-3p, evidencing failure in functionalization.

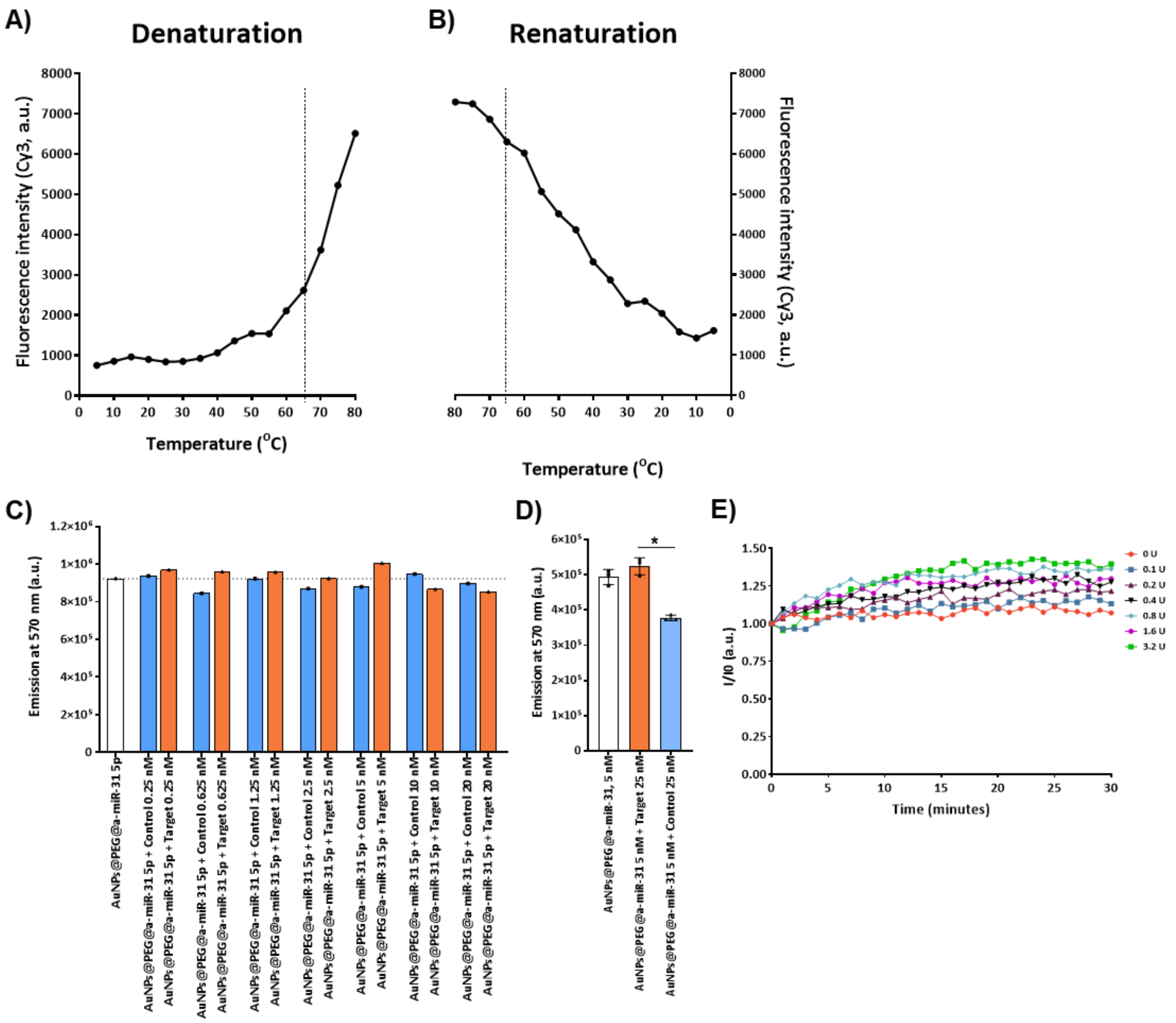

Figure S2 – Evaluation of the functionality and stability of the molecular beacon attached to the AuNPs@PEG@α-miR-31-5p nanobeacone. (A) Melting curve: denaturation – Cy3 emission of AuNPs@PEG@α-miR-31-5p obtained by increasing temperature, showing the increase in fluorescence emission when the molecular beacon's hairpin is in the 'on' state. (B) Melting curve: renaturation – Cy3 emission of AuNPs@PEG@α-miR-31-5p obtained by decreasing temperature, showing the decrease in fluorescence emission when the molecular beacon's hairpin is in the 'off' state. The vertical dashed line corresponds to the melting temperature (53.1 ºC) of the molecular beacon. (C) Cy3 emission obtained in the interaction of AuNPs@PEG@α-miR-31-5p (1 nM) with its miRNA target, miR-31-5p (in orange, different concentrations), compared to nanobeacon alone (in white) and to interaction with an irrelevant sequence (in blue). The horizontal dashed line represents the fluorescence level of the nanobeacon alone. (D) Cy3 emission obtained in the interaction of AuNPs@PEG@α-miR-31-5p (5 nM) with its miRNA target, miR-31-5p (in orange, 25 nM), compared to nanobeacon alone (in white) and to interaction with an irrelevant sequence (in blue, 25 nM). (E) Evaluation of the interaction of AuNPs@PEG@α-miR-31-5p in the presence of different concentrations of RNase, evidencing a dose-dependent degradation of the molecular beacon's hairpin.

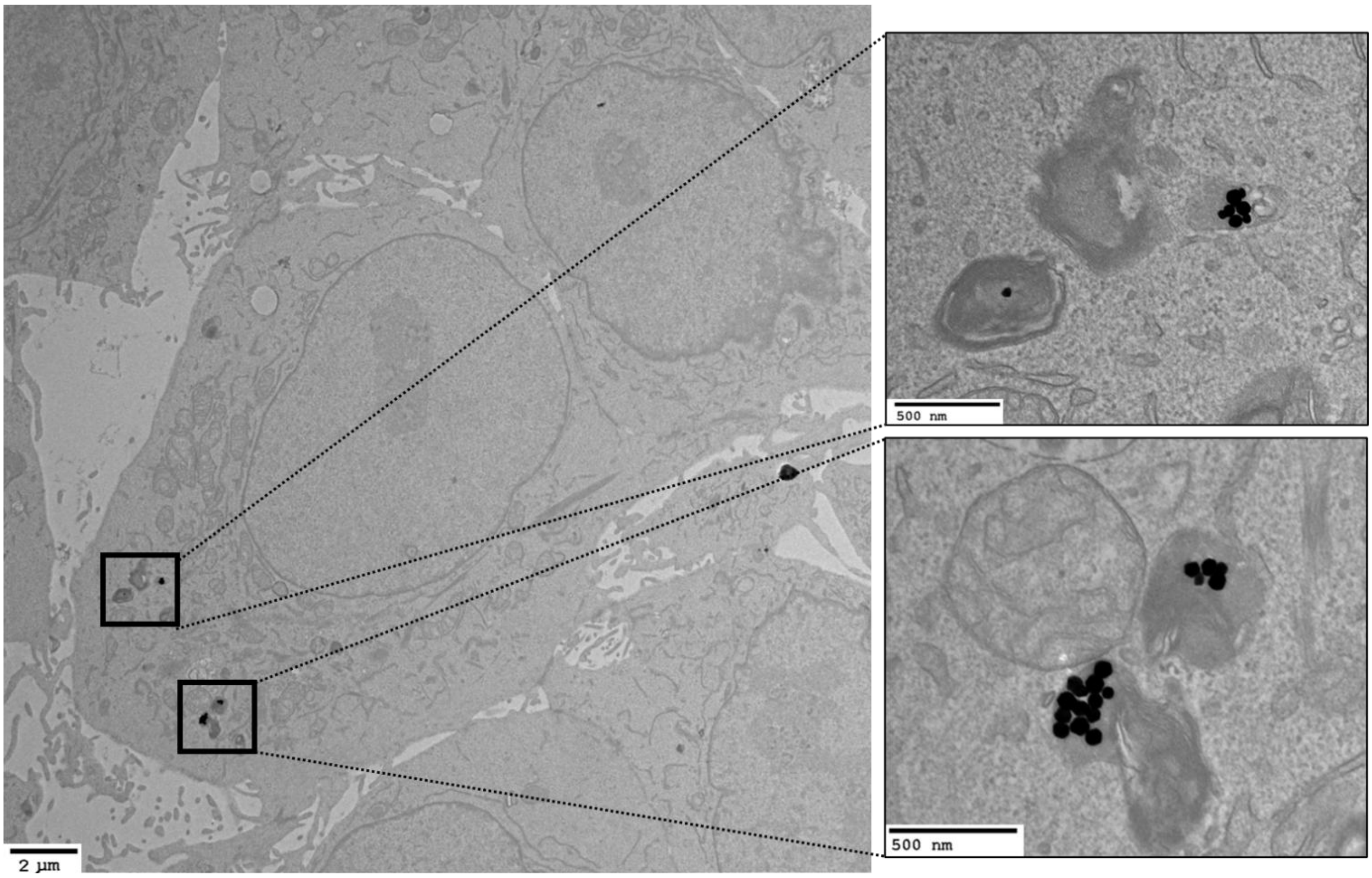

Figure S3 – Internalization and cellular localization of PEGylated AuNPs. TEM photographs of A549-Luc at 72 h after treatment with AuNPs@PEG (10 nM), revealing electron-dense Au cores in membrane-bound vesicles (left, low magnification; right, high-magnification insets) consistent with late endosome/lysosome compartments. Scale bar (left): 2 µm; right insets: 500 nm.

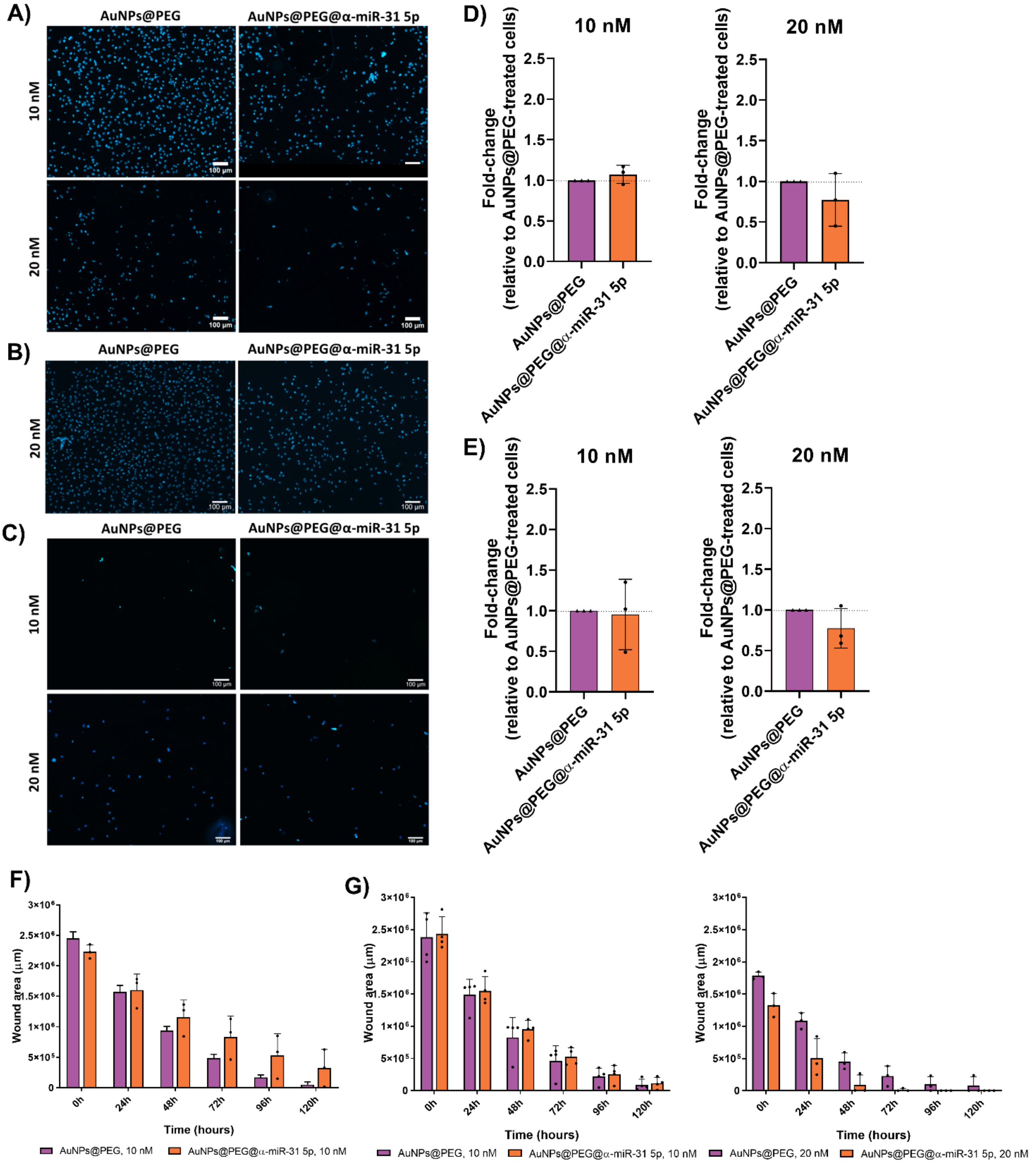

Figure S4 – Evaluation of the effects of the AuNPs@PEG and AuNPs@PEG@α-miR-31 5p nanoconjugates on the migration and invasion capacities of lung cancer cells. (A) Representative pictures of the nuclei of invasive A549-Luc cells, stained with DAPI, after 72 h of pre-treatment with AuNPs@PEG (left) or nanobeacons (right) at 10 nM (top) or 20 nM (bottom) and transwell invasion for 24 h. (B) Representative pictures of the nuclei of invasive A549-Luc cells, stained with DAPI, after 120 h of pre-treatment with AuNPs@PEG (left) or nanobeacons (right) at 20 nM and transwell invasion for 24 h. (C) Representative pictures of the nuclei of invasive RERF-LC-Sq1 CMV-Luc cells, stained with DAPI, after 72 h of pre-treatment with AuNPs@PEG (left) or nanobeacons (right) at 10 nM (top) or 20 nM (bottom) and transwell invasion for 24 h. Scale bars = 100 µm. (D) Quantification of the migration of A549-Luc cells, after pre-treatment for 72 h with AuNPs@PEG (purple) or nanobeacons (orange) and transwell migration for 24 h, using concentrations of 10 nM (left) and 20 nM (right), in fold-change relative to cells treated with AuNPs@PEG. (E) Quantification of the migration of RERF-LC-Sq1 CMV-Luc cells, after pre-treatment for 72 h with AuNPs@PEG (purple) or nanobeacons (orange) and transwell

migration for 24 h, using concentrations of 10 nM (left) and 20 nM (right), in fold-change relative to cells treated with AuNPs@PEG. (F) Quantification of the wound area (µm) on A549-Luc cells, followed for 120 h after the wound (t = 0 h), after pre-treatment for 48 h with AuNPs@PEG (purple) or nanobeacons (orange), using a concentration of 10 nM. (G) Quantification of the wound area (µm) on A549-Luc cells, followed for 120 h after the wound (t = 0 h), after pre-treatment for 72 h with AuNPs@PEG (purple) or nanobeacons (orange), using concentration of 10 nM (left) or 20 nM (right).

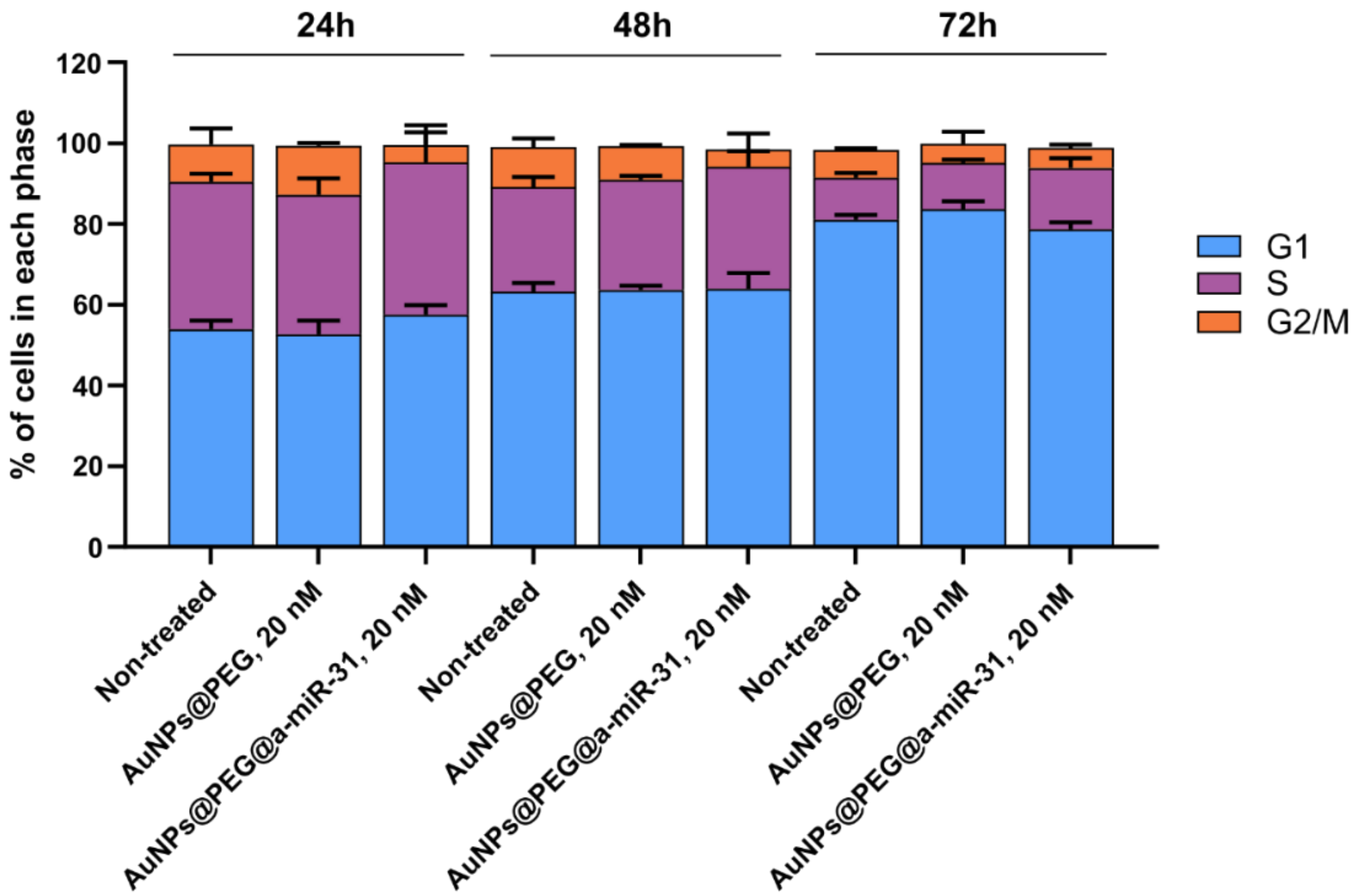

Figure S5 – Evaluation of the effects of the AuNPs@PEG and AuNPs@PEG@α-miR-31 5p nanobeacons on the cell cycle of A549-Luc cells. Flow cytometry was used to evaluate the cells after treatment for 24, 48, and 72 hours, using a concentration of 20 nM. The G1 phase is represented in blue, the M phase is represented in purple, and the G2/M phase is represented in orange. An arrest in the G1 phase, visible as an increase of the percentage of cells at this phase, is observed as the time exposure to nanobeacons increases.

Table S3 – Differentially expressed genes (upregulated) obtained upon RNA extraction and mRNA sequencing of A54-Luc cells treated with AuNPs@PEG@α-miR-31-5p, relative to A549-Luc cells treated with AuNPs@PEG for 72 h.

Gene ID	Gene Name	Adjusted p-value (padj)	log_2 (Fold Change)
ENSG00000152377	*SPOCK1*	$3.55x10^{-14}$	2.983340667
ENSG00000135842	*FAM129A*	$3.29x10^{-13}$	1.264294162
ENSG00000130635	*COL5A1*	$1.98x10^{-11}$	1.933099816
ENSG00000238266	*LINC00707*	$9.93x10^{-11}$	1.547030871
ENSG00000128564	*VGF*	$5.37x10^{-10}$	1.678941972
ENSG00000144583	*MARCH4*	$3.41x10^{-9}$	2.610480549
ENSG00000188293	*IGFL1*	$2.00x10^{-8}$	3.194903938
ENSG00000129353	*SLC44A2*	$7.59x10^{-7}$	1.053260382
ENSG00000104967	*NOVA2*	$1.26x10^{-6}$	1.265403984
ENSG00000124225	*PMEPA1*	$2.96x10^{-6}$	1.27087012
ENSG00000103647	*CORO2B*	$3.44x10^{-5}$	1.913558737
ENSG00000167332	*OR51E2*	$5.20x10^{-5}$	1.052169775
ENSG00000213626	*LBH*	$5.59x10^{-5}$	1.250346265
ENSG00000163202	*LCE3D*	$9.58x10^{-5}$	2.907236959
ENSG00000160201	*U2AF1*	$1.25x10^{-4}$	2.302638425
ENSG00000106366	*SERPINE1*	$1.63x10^{-4}$	1.300610638
ENSG00000100867	*DHRS2*	$1.74x10^{-4}$	1.404782164
ENSG00000185966	*LCE3E*	$3.33x10^{-4}$	4.602212657
ENSG00000205572	*SERF1B*	$4.18x10^{-4}$	1.6241936
ENSG00000155816	*FMN2*	$9.79x10^{-4}$	1.052806117
ENSG00000232024	*LSM12P1*	$1.72x10^{-3}$	1.142510017
ENSG00000116254	*CHD5*	$1.86x10^{-3}$	1.333499645
ENSG00000091137	*SLC26A4*	$2.30x10^{-3}$	5.448702198
ENSG00000095752	*IL11*	$2.59x10^{-3}$	1.073686844
ENSG00000117115	*PADI2*	$2.75x10^{-3}$	1.081716655
ENSG00000171368	*TPPP*	$3.50x10^{-3}$	1.207464503
ENSG00000145002	*FAM86B2*	$4.44x10^{-3}$	1.558248325
ENSG00000185565	*LSAMP*	$7.08x10^{-3}$	1.907538825
ENSG00000184574	*LPAR5*	$7.51x10^{-3}$	1.306235732
ENSG00000074047	*GLI2*	$7.68x10^{-3}$	1.28821712
ENSG00000185567	*AHNAK2*	$7.68x10^{-3}$	1.301927483
ENSG00000078725	*BRINP1*	$8.46x10^{-3}$	1.402895427
ENSG00000149571	*KIRREL3*	$1.14x10^{-2}$	1.056040965
ENSG00000258711	*AL358334.2*	$1.18x10^{-2}$	1.086413212
ENSG00000171246	*NPTX1*	$1.26x10^{-2}$	1.975979787
ENSG00000100626	*GALNT16*	$1.27x10^{-2}$	1.276648729
ENSG00000268940	*CT45A1*	$1.29x10^{-2}$	4.034807271
ENSG00000225675	*LINC01771*	$1.30x10^{-2}$	4.003040829
ENSG00000233818	*AP000695.2*	$1.64x10^{-2}$	1.968674638
ENSG00000180785	*OR51E1*	$2.03x10^{-2}$	1.641549676
ENSG00000179242	*CDH4*	$2.37x10^{-2}$	1.057900736
ENSG00000154764	*WNT7A*	$2.65x10^{-2}$	6.740868863
ENSG00000272316	*AL021368.2*	$3.18x10^{-2}$	1.004064284
ENSG00000110427	*KIAA1549L*	$3.71x10^{-2}$	1.540937904
ENSG00000204767	*INSYN2B*	$4.50x10^{-2}$	3.452830881
ENSG00000106799	*TGFBR1*	$4.61x10^{-2}$	1.080209833

Table S4 – Differentially expressed genes (downregulated) obtained upon RNA extraction and mRNA sequencing of A54-Luc cells treated with AuNPs@PEG@α-miR-31-5p, relative to A549-Luc cells treated with AuNPs@PEG for 72 h.

Gene ID	Gene Name	Adjusted p-value (padj)	log_2 (Fold Change)
ENSG00000166523	*CLEC4E*	$4.76x10^{-45}$	-2.708948066
ENSG00000163739	*CXCL1*	$4.72x10^{-34}$	-2.482698035
ENSG00000108691	*CCL2*	$1.10x10^{-31}$	-2.195856963
ENSG00000160326	*SLC2A6*	$3.71x10^{-27}$	-2.109632846
ENSG00000169429	*CXCL8*	$3.45x10^{-26}$	-2.154676764
ENSG00000124102	*PI3*	$6.96x10^{-23}$	-2.580810129
ENSG00000100906	*NFKBIA*	$1.77x10^{-19}$	-1.439087225
ENSG00000112096	*SOD2*	$7.29x10^{-17}$	-1.367841831
ENSG00000134827	*TCN1*	$2.88x10^{-13}$	-1.424046904
ENSG00000115009	*CCL20*	$2.88x10^{-13}$	-2.231852931
ENSG00000007952	*NOX1*	$1.14x10^{-10}$	-1.974737857
ENSG00000004468	*CD38*	$1.56x10^{-10}$	-1.446613515
ENSG00000163734	*CXCL3*	$1.83x10^{-10}$	-1.554774964
ENSG00000116133	*DHCR24*	$4.06x10^{-10}$	-1.054036587
ENSG00000184371	*CSF1*	$2.70x10^{-9}$	-1.086642509
ENSG00000227507	*LTB*	$4.02x10^{-9}$	-2.725441813
ENSG00000162896	*PIGR*	$7.55x10^{-9}$	-3.775606951
ENSG00000163131	*CTSS*	$1.83x10^{-8}$	-1.189005176
ENSG00000135127	*BICDL1*	$1.83x10^{-8}$	-1.048658081
ENSG00000081041	*CXCL2*	$2.99x10^{-8}$	-1.564295965
ENSG00000023445	*BIRC3*	$4.12x10^{-8}$	-1.499669791
ENSG00000077150	*NFKB2*	$4.75x10^{-8}$	-1.345491938
ENSG00000174837	*ADGRE1*	$1.68x10^{-7}$	-1.854103284
ENSG00000108342	*CSF3*	$7.90x10^{-7}$	-3.849090077
ENSG00000060566	*CREB3L3*	$8.42x10^{-7}$	-2.11550285
ENSG00000008517	*IL32*	$1.57x10^{-6}$	-1.924796949
ENSG00000125999	*BPIFB1*	$1.72x10^{-6}$	-2.043696695
ENSG00000096006	*CRISP3*	$2.45x10^{-6}$	-1.235332268
ENSG00000116661	*FBXO2*	$3.18x10^{-6}$	-2.226681446
ENSG00000169403	*PTAFR*	$5.38x10^{-6}$	-1.296817506
ENSG00000173432	*SAA1*	$6.26x10^{-6}$	-2.901124685
ENSG00000257335	*MGAM*	$7.61x10^{-6}$	-1.299792017
ENSG00000166920	*C15orf48*	$8.36x10^{-6}$	-2.258496954
ENSG00000166396	*SERPINB7*	$8.94x10^{-6}$	-1.473957216
ENSG00000237988	*OR2I1P*	$1.98x10^{-5}$	-2.552164108
ENSG00000104856	*RELB*	$1.98x10^{-5}$	-1.241003888
ENSG00000169242	*EFNA1*	$2.47x10^{-5}$	-1.171393235
ENSG00000148346	*LCN2*	$2.55x10^{-5}$	-1.579553724
ENSG00000049249	*TNFRSF9*	$2.80x10^{-5}$	-1.071590991
ENSG00000090339	*ICAM1*	$5.86x10^{-5}$	-2.648517972
ENSG00000161270	*NPHS1*	$8.72x10^{-5}$	-1.709648822
ENSG00000187037	*GPR141*	$9.58x10^{-5}$	-1.83014356
ENSG00000133661	*SFTPD*	$9.94x10^{-5}$	-1.69525528
ENSG00000170075	*GPR37L1*	$1.14x10^{-4}$	-2.286024505
ENSG00000226054	*MEMO1P1*	$1.25x10^{-4}$	-2.290472836
ENSG00000138207	*RBP4*	$1.37x10^{-4}$	-1.207360215
ENSG00000138109	*CYP2C9*	$1.54x10^{-4}$	-3.084218087
ENSG00000243566	*UPK3B*	$2.15x10^{-4}$	-1.311057178
ENSG00000243649	*CFB*	$2.18x10^{-4}$	-1.290916541
ENSG00000263528	*IKBKE*	$2.33x10^{-4}$	-1.040584912
ENSG00000105246	*EBI3*	$2.37x10^{-4}$	-1.290444717
ENSG00000159403	*C1R*	$2.63x10^{-4}$	-1.017353797
ENSG00000283413	*AC008703.1*	$3.33x10^{-4}$	-1.494346595

ENSG00000111801	*BTN3A3*	$4.11x10^{-4}$	-1.178375122
ENSG00000162645	*GBP2*	$4.24x10^{-4}$	-1.090886202
ENSG00000137440	*FGFBP1*	$4.36x10^{-4}$	-1.6036896
ENSG00000130558	*OLFM1*	$6.77x10^{-4}$	-1.001826476
ENSG00000268621	*IGFL2-AS1*	$9.28x10^{-4}$	-1.031985938
ENSG00000105499	*PLA2G4C*	$1.08x10^{-3}$	-1.259085164
ENSG00000231574	*LINC02015*	$1.08x10^{-3}$	-2.416315273
ENSG00000152766	*ANKRD22*	$1.22x10^{-3}$	-1.280342254
ENSG00000124107	*SLPI*	$1.27x10^{-3}$	-1.643596541
ENSG00000215841	*AP002761.1*	$1.27x10^{-3}$	-8.223762953
ENSG00000103313	*MEFV*	$1.69x10^{-3}$	-2.295111784
ENSG00000146232	*NFKBIE*	$1.72x10^{-3}$	-1.239720511
ENSG00000170458	*CD14*	$2.11x10^{-3}$	-1.409128233
ENSG00000188488	*SERPINA5*	$2.11x10^{-3}$	-1.715270327
ENSG00000189058	*APOD*	$2.47x10^{-3}$	-1.809949834
ENSG00000064886	*CHI3L2*	$2.47x10^{-3}$	-1.716645587
ENSG00000200428	*RF00019*	$4.47x10^{-3}$	-8.208884552
ENSG00000196542	*SPTSSB*	$5.12x10^{-3}$	-1.347280511
ENSG00000225940	*C5orf67*	$5.51x10^{-3}$	-3.530553248
ENSG00000118322	*ATP10B*	$6.25x10^{-3}$	-1.252156411
ENSG00000281655	*AP000851.2*	$7.12x10^{-3}$	-2.63368958
ENSG00000276980	*AC008760.2*	$7.18x10^{-3}$	-1.818698975
ENSG00000164825	*DEFB1*	$7.44x10^{-3}$	-1.326707338
ENSG00000168065	*SLC22A11*	$7.68x10^{-3}$	-1.716677013
ENSG00000137648	*TMPRSS4*	$7.68x10^{-3}$	-1.154766771
ENSG00000261040	*WFDC21P*	$7.79x10^{-3}$	-2.385173216
ENSG00000139445	*FOXN4*	$7.93x10^{-3}$	-1.54063812
ENSG00000156414	*TDRD9*	$9.15x10^{-3}$	-8.054051924
ENSG00000255860	*AP000812.2*	$9.59x10^{-3}$	-4.161522097
ENSG00000125780	*TGM3*	$1.01x10^{-2}$	-1.191845634
ENSG00000134070	*IRAK2*	$1.08x10^{-2}$	-1.159185096
ENSG00000100170	*SLC5A1*	$1.19x10^{-2}$	-2.644895502
ENSG00000160013	*PTGIR*	$1.21x10^{-2}$	-2.263767407
ENSG00000170373	*CST1*	$1.22x10^{-2}$	-1.2523881
ENSG00000275302	*CCL4*	$1.26x10^{-2}$	-5.804894538
ENSG00000197880	*MDS2*	$1.30x10^{-2}$	-2.433413496
ENSG00000139547	*RDH16*	$1.31x10^{-2}$	-1.344909426
ENSG00000080910	*CFHR2*	$1.33x10^{-2}$	-2.742414118
ENSG00000167617	*CDC42EP5*	$1.38x10^{-2}$	-1.968169334
ENSG00000162552	*WNT4*	$1.49x10^{-2}$	-1.36854902
ENSG00000073756	*PTGS2*	$1.49x10^{-2}$	-1.047744264
ENSG00000225762	*LINC01389*	$1.64x10^{-2}$	-1.553449162
ENSG00000163121	*NEURL3*	$1.74x10^{-2}$	-1.626071296
ENSG00000106302	*HYAL4*	$1.87x10^{-2}$	-2.03865071
ENSG00000137731	*FXYD2*	$2.15x10^{-2}$	-1.094632114
ENSG00000187134	*AKR1C1*	$2.47x10^{-2}$	-1.069199322
ENSG00000137474	*MYO7A*	$2.65x10^{-2}$	-1.01477072
ENSG00000167207	*NOD2*	$2.66x10^{-2}$	-2.864352698
ENSG00000163220	*S100A9*	$2.66x10^{-2}$	-1.565429729
ENSG00000197408	*CYP2B6*	$2.74x10^{-2}$	-1.505107228
ENSG00000135472	*FAIM2*	$2.82x10^{-2}$	-1.263347256
ENSG00000162888	*C1orf147*	$2.86x10^{-2}$	-2.410704293
ENSG00000102760	*RGCC*	$2.86x10^{-2}$	-1.336932344
ENSG00000232810	*TNF*	$2.87x10^{-2}$	-3.390786859
ENSG00000196154	*S100A4*	$2.89x10^{-2}$	-1.546758898
ENSG00000186198	*SLC51B*	$3.02x10^{-2}$	-1.285723243
ENSG00000185186	*LINC00313*	$3.03x10^{-2}$	-1.855345936
ENSG00000266378	*AC005224.3*	$3.26x10^{-2}$	-7.091629094

ENSG00000088002	*SULT2B1*	$3.27x10^{-2}$	-1.102074611
ENSG00000142449	*FBN3*	$3.55x10^{-2}$	-3.626543195
ENSG00000177455	*CD19*	$3.62x10^{-2}$	-7.574331722
ENSG00000278106	*AF254983.2*	$3.62x10^{-2}$	-6.724696515
ENSG00000183324	*REC114*	$3.64x10^{-2}$	-3.734276939
ENSG00000178199	*ZC3H12D*	$3.71x10^{-2}$	-1.866627801
ENSG00000204257	*HLA-DMA*	$3.95x10^{-2}$	-1.142786157
ENSG00000227154	*AL022721.1*	$3.99x10^{-2}$	-7.126211087
ENSG00000257017	*HP*	$4.12x10^{-2}$	-2.374742356
ENSG00000168350	*DEGS2*	$4.30x10^{-2}$	-1.042698113
ENSG00000163218	*PGLYRP4*	$4.31x10^{-2}$	-3.847087052
ENSG00000162692	*VCAM1*	$4.50x10^{-2}$	-1.632949772
ENSG00000110799	*VWF*	$4.50x10^{-2}$	-3.775361919
ENSG00000258498	*DIO3OS*	$4.60x10^{-2}$	-1.231545651
ENSG00000285533	*AP001362.2*	$4.91x10^{-2}$	-1.042899143
ENSG00000280649	*AC245100.8*	$4.97x10^{-2}$	-1.19743454

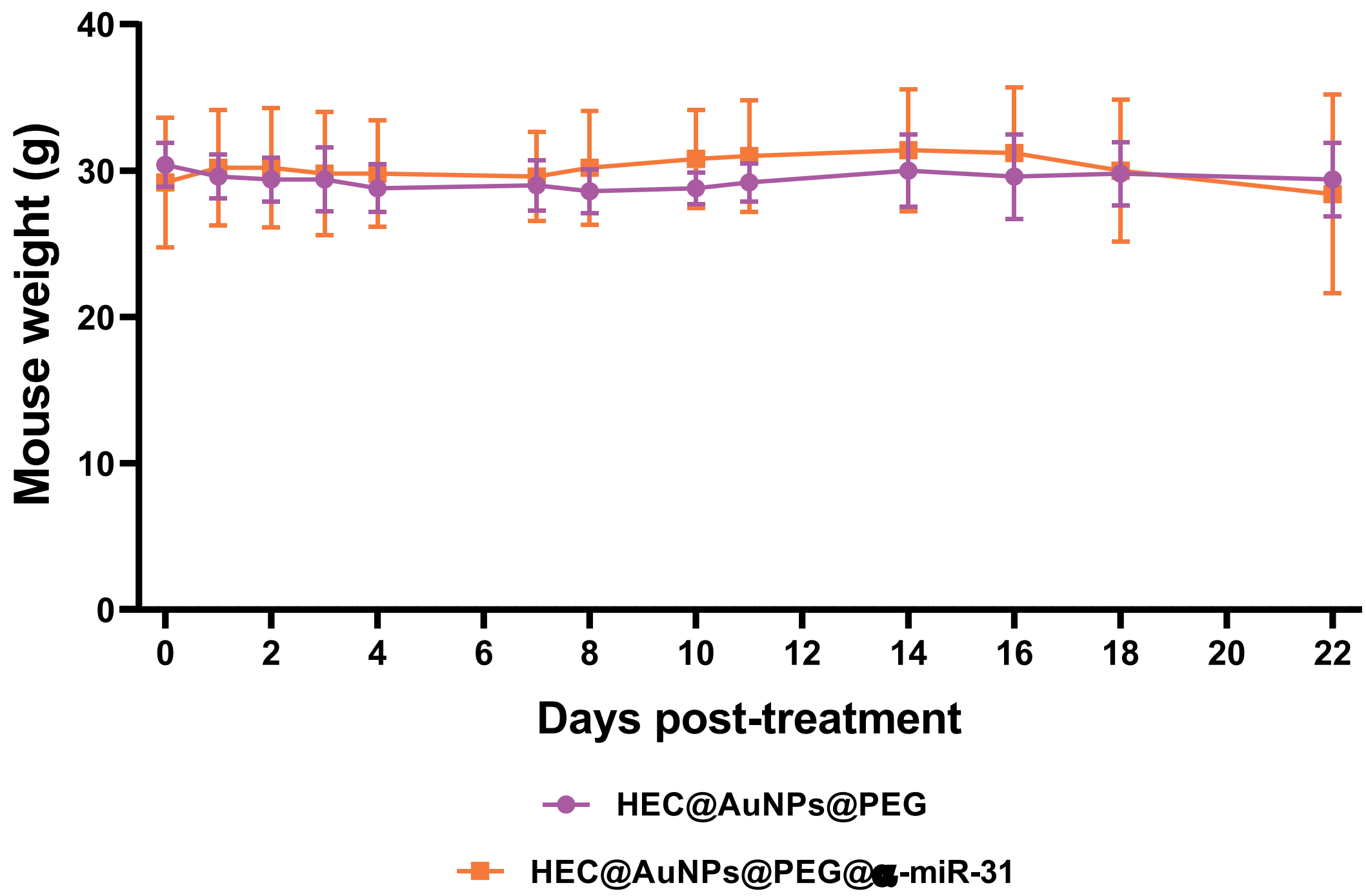

Figure S6 – Evaluation of potential toxicity of HEC@AuNPs@PEG (purple) and HEC@AuNPs@PEG@α-miR-31-5p (orange) *in vivo.* Mice weight was monitored for 22 days post-treatment and no significant alterations were observed throughout the experiment, showing the biocompatibility of the nanobeacons.

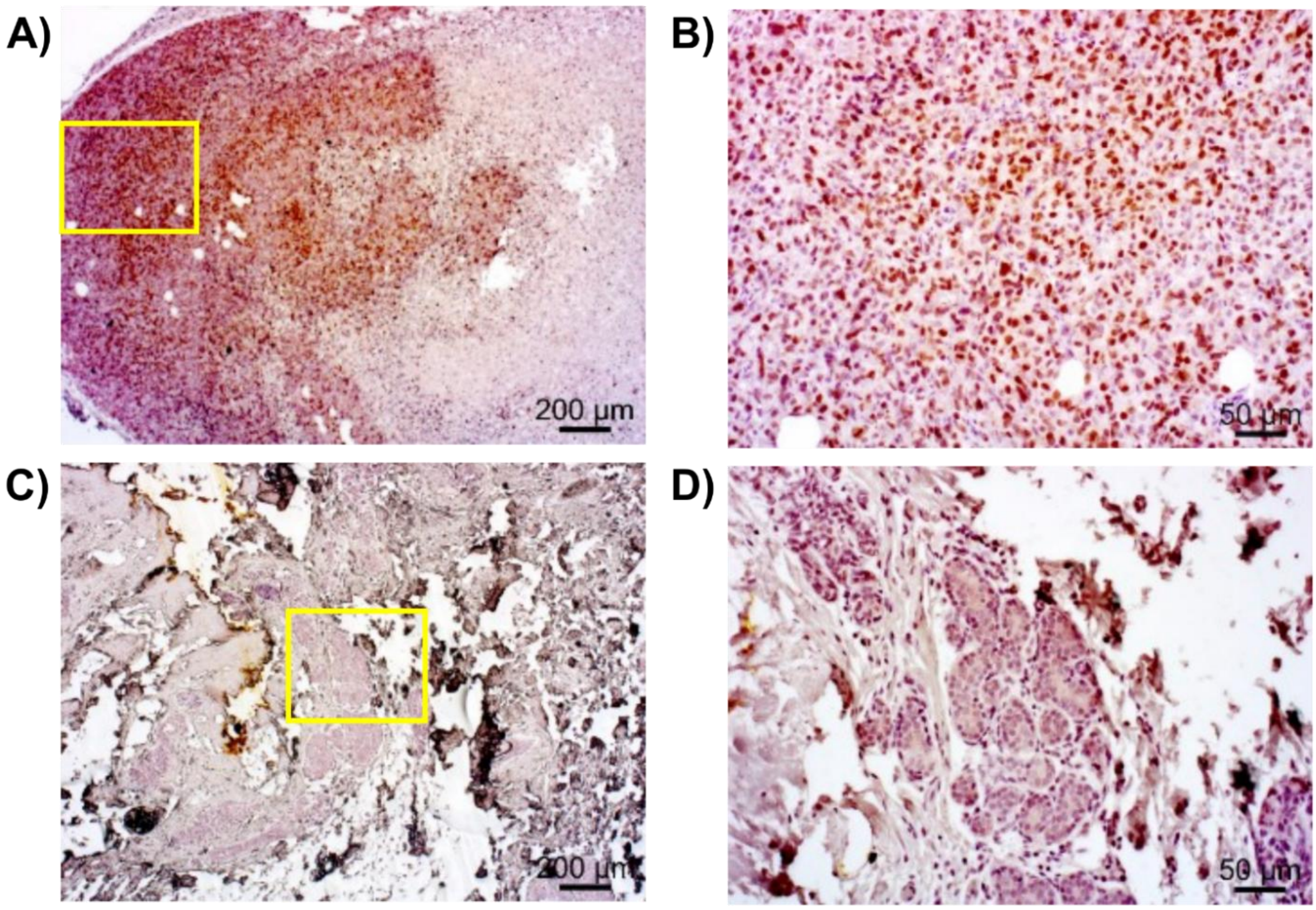

Figure S7 – Controls used for Ki-67 IHC staining. (A) Section of a highly proliferative breast cancer cell line (MDA-MB-231), showing positive Ki-67 staining as shown by the brown nuclei. The yellow rectangle highlights the region shown as a zoomed image in (B). The scale bar corresponds to 200 µm. (B) Magnification of the region shown in (A), showing the brown nuclei staining well spread throughout the tissue. The scale bar corresponds to 50 µm. (C) Section of a low proliferative breast tissue, showing low Ki-67 positivity, as shown by the absence of brown nuclei. The scale bar corresponds to 200 µm. (D) Magnification of the region shown in (C), showing very few brown nuclei stained. The scale bar corresponds to 50 µm.

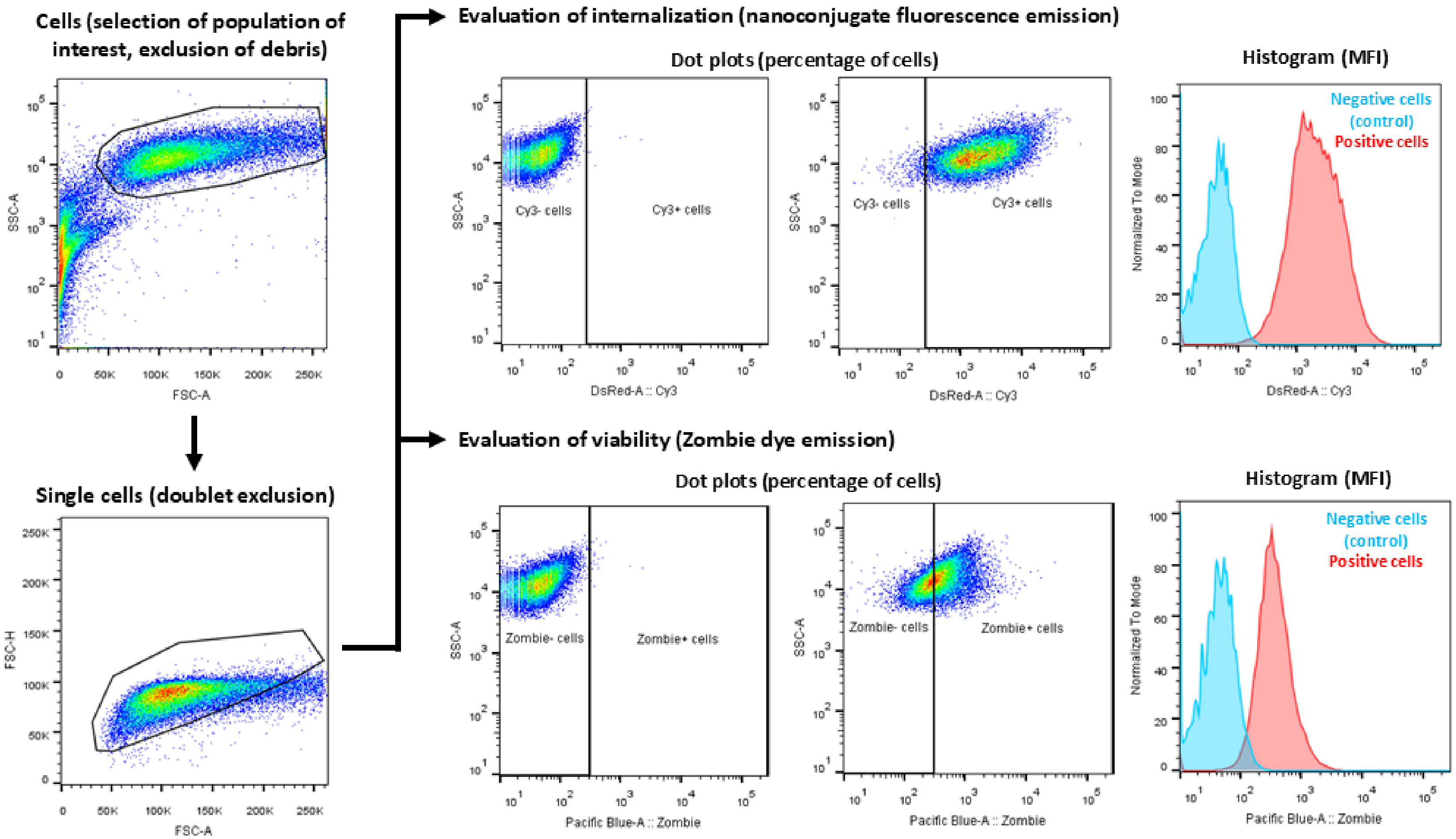

Figure S8 - General gating strategy used for acquisition and analysis of flow cytometry data regarding viability and internalization assays. The population of interest was selected, doublets were removed and internalization and viability were evaluated using dot plots to obtain information regarding the percentage of negative and positive cells, and histograms to obtain information regarding the fluorescence emission, MFI.

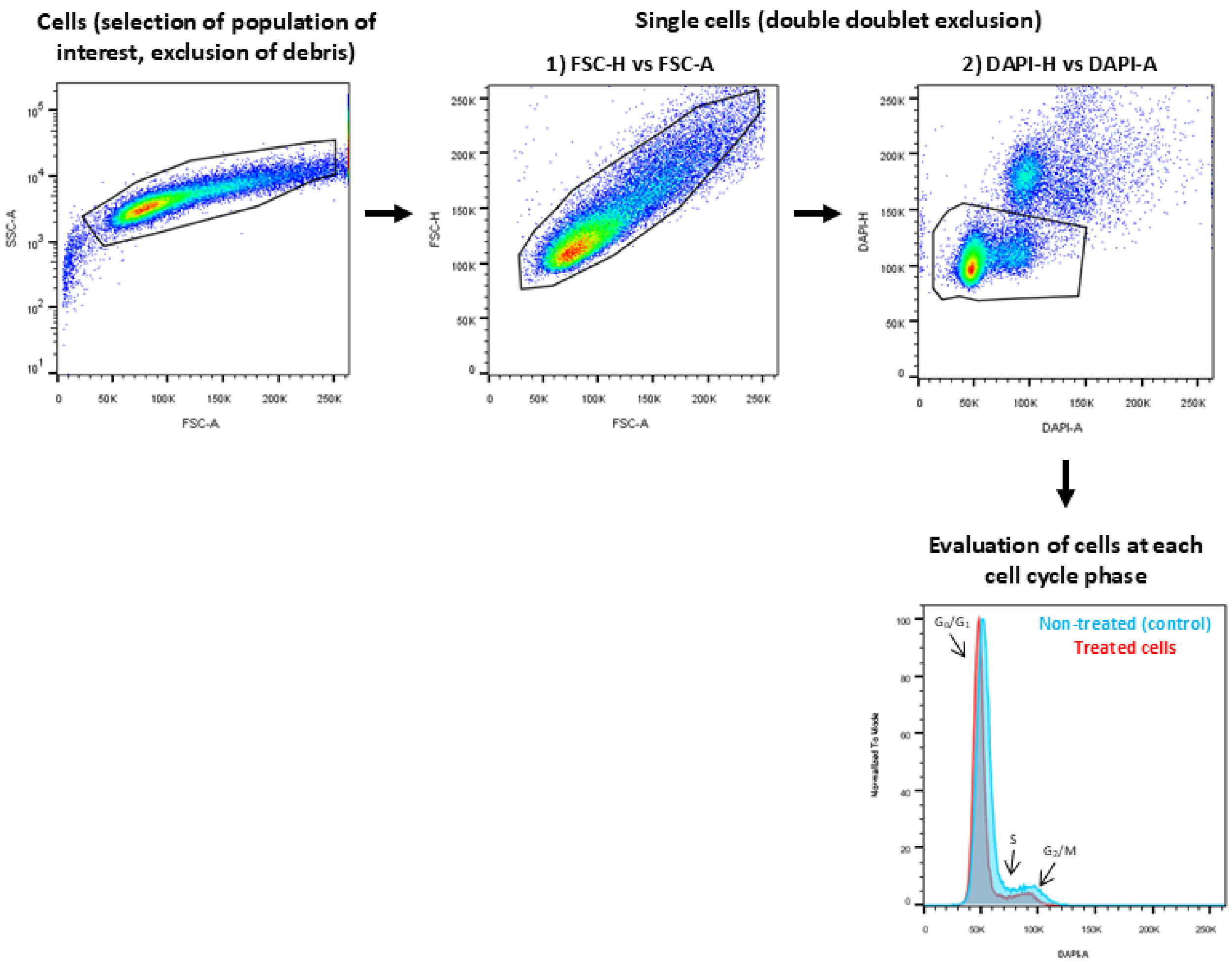

Figure S9 - General gating strategy used for acquisition and analysis of flow cytometry data regarding cell cycle assays. The population of interest was selected, doublets were removed, and the percentage of cells at each phase of the cell cycle was evaluated using the models available in the FlowJo software.

Supplementary Methods

1. Complementary characterization of the gold nanobeacons

1.1. Hairpin reversible denaturation-renaturation

To evaluate the capacity of the molecular beacon' hairpin to open and close according to a temperature stimuli upon functionalization to the AuNPs, gold nanobeacons were added, in triplicate, in the wells of a MicroAmp Optical 96-Well Reaction Plate (Thermofisher Scientific #N8010560). The plate was added to a thermocycler (QuantStudio 5) and the samples were subjected to an increasing range of temperatures, starting at 5ºC and ending at 80ºC, while collecting fluorescence emission (Cy3) data. Upon reaching 80ºC, the same process was performed by decreasing the temperatures back to 5ºC. The fluorescence emission was plotted in graphs for better visualization.

1.2. Target hybridization

To evaluate the recognition of the miRNA target by the gold nanobeacons, oligonucleotides matching the target miRNA sequence (hsa-miR-31-5p) and a control sequence with no biological activity were purchased from STABVida (custom made). Stock solutions were prepared in nuclease-free water at a concentration of 1000 nM and stored at -80ºC. The nanobeacons were incubated alone, in triplicate, at concentrations of 1 nM and 5 nM, for 5 minutes on a heat block at 90ºC, to promote the opening of the molecular beacons at the surface of the AuNPs. Then, target/control sequences were added to reach final concentrations of 0.25, 0.625, 1.25, 2.5, 5, 10, 20 and 25 nM, incubated for 5 minutes and then samples were transferred to ice, to promote the closing of the molecular beacons, and left to cool for 10 minutes. Samples were then transferred to a 96-well plate, fluorescence emission data (Cy3) was acquired in a Spectramax i3X plate reader (Molecular Devices), using the software SoftMax Pro 7, and results were plotted in graphs for better visualization.

1.3. Interaction with RNase

The stability of the gold nanobeacons in the presence of RNase was evaluated. To do this, solutions of 1 nM of gold nanobeacons were prepared in nuclease-free water and then incubated, in triplicate, in 96-well plates, with RNase at the following concentrations: 0, 0.2, 0.2, 0.4, 0.8, 1.6 and 3.2 U, for 30 minutes at 37ºC. Fluorescence emission data was acquired every minute in a Spectramax i3X plate reader (Molecular Devices), using the software SoftMax Pro 7. The emission at each timepoint was normalized to the time = 0 h (I/I_0) and data were plotted in graphs for better visualization.

2. Complementary functional analysis of the gold nanobeacons

2.1. Cell cycle

The effects of the AuNPs@PEG and gold nanobeacons on the cell cycle were evaluated by analyzing the DNA content of cells using flow cytometry. A549-Luc cells were plated (200 000 cells/well, 1 ml/well) in serum-free medium on the wells of 24-well plates and allowed to attach and synchronize by starvation for 24 hours. The medium was exchanged and the AuNPs@PEG/nanobeacons were added (20 nM) and incubated with the cells for 24, 48 and 72 h. Cells were detached and washed. The supernatant was discarded, and the pellet was resuspended and vortexed in 1 ml of cold ethanol (70% v/v) to fix the cells. The samples were stored at -20ºC for at least 1 hour and then washed to remove the ethanol. The pelleted cells were resuspended in 200 µl of DAPI working solution (10 µg/ml DAPI and 100 mg/ml RNase) and incubated (30 minutes, RT, dark), and transferred to 5 ml tubes. Data was acquired using a FACS Canto II cytometer (BD Biosciences) and using the BD FACS Diva Software (BD Biosciences) by collecting at least 30 000 cells in the 'single cells' gate. Analysis was performed in the software FlowJo (V10, Tree Star, Inc.) and using the Dean-Jett-Fox model.

2.2. miR-31 quantification by qRT-PCR

Quantification of the levels of miR-31-5p using qRT-PCR were performed as described in the manuscript, using the following reagents and reaction volumes:

Table S5 - Components used on the conversion of RNA to cDNA, and respective volumes, in microliters, per sample.

Component	Volume per reaction (µl)
10x RT Buffer	2
25x dNTP mix (100 mM)	0.8
10x RT random primers	2
Multiscribe reverse transcriptase	1
RNase inhibitor	1
miR-31 RT primer 5x (target)	3
RNU6B RT primer 5x (endogenous control)	3
Nuclease-free H_2O	1
Total per reaction	13.8

Table S6 - Components used on the qRT-PCR reaction and respective volumes, in microliters, per sample, for the two master mixes prepared.

**Components – Master mix 1 (target miRNA)**	**Volume per reaction (µl)**
Taqman Fast Advanced Master Mix	10
Taqman Advanced miRNA Assay 20x (hsa-miR-31)	1
Nuclease-free H_2O	4
Total per reaction	15

**Components – Master mix 2 (endogenous control)**	**Volume per reaction (µl)**
Taqman Fast Advanced Master Mix	10
Taqman Advanced miRNA Assay 20x (RNU6B)	1
Nuclease-free H_2O	4
Total per reaction	15
